# Supplementary material for: Iron overload induced death of osteoblasts in vitro: involvement of the mitochondrial apoptotic pathway
Source: PeerJ. 2016 Nov 8;4:e2611. doi: 10.7717/peerj.2611 (PMC5103817; doi:10.7717/peerj.2611)
Supplement: Data S1 [file peerj-04-2611-s001.pdf]

Figure 1 Cytotoxic effects of iron on the viability of osteoblasts.

24h

	Control	FAC 25	FAC 50	FAC 100	FAC 200
Group1	1.061	1.061	1.051	1.056	1.042
	1.005	1.004	1.086	1.002	1.001
	1.029	0.999	1.075	1.067	0.995
Group2	1.053	0.958	1.141	1.009	1.011
	1.075	1.012	0.974	1.017	1.068
	1.136	0.967	0.869	1.15	1.148
Group3	1.032	1.056	1.105	0.943	0.908
	0.964	1.092	1.006	0.951	0.987
	0.978	1.101	0.957	0.993	0.981

72h

	Control	FAC 25	FAC 50	FAC 100	FAC 200
Group1	1.47	1.471	1.479	1.336	1.241
	1.473	1.482	1.392	1.275	1.195
	1.388	1.394	1.506	1.318	1.228
Group2	1.485	1.488	1.498	1.353	1.251
	1.487	1.488	1.493	1.346	1.237
	1.498	1.511	1.527	1.375	1.246
Group3	1.493	1.518	1.462	1.361	1.262
	1.539	1.529	1.482	1.282	1.242
	1.496	1.535	1.512	1.332	1.281

120h

	Control	FAC 25	FAC 50	FAC 100	FAC 200
Group1	1.747	1.639	1.538	1.061	0.926
	1.776	1.599	1.479	1.101	0.889
	1.836	1.606	1.521	1.121	0.901
Group2	1.672	1.749	1.456	1.119	0.869
	1.635	1.745	1.421	1.048	0.798
	1.561	1.768	1.378	1.082	0.827
Group3	1.764	1.722	1.435	0.902	0.753
	1.719	1.745	1.405	0.985	0.721
	1.665	1.688	1.443	0.967	0.727

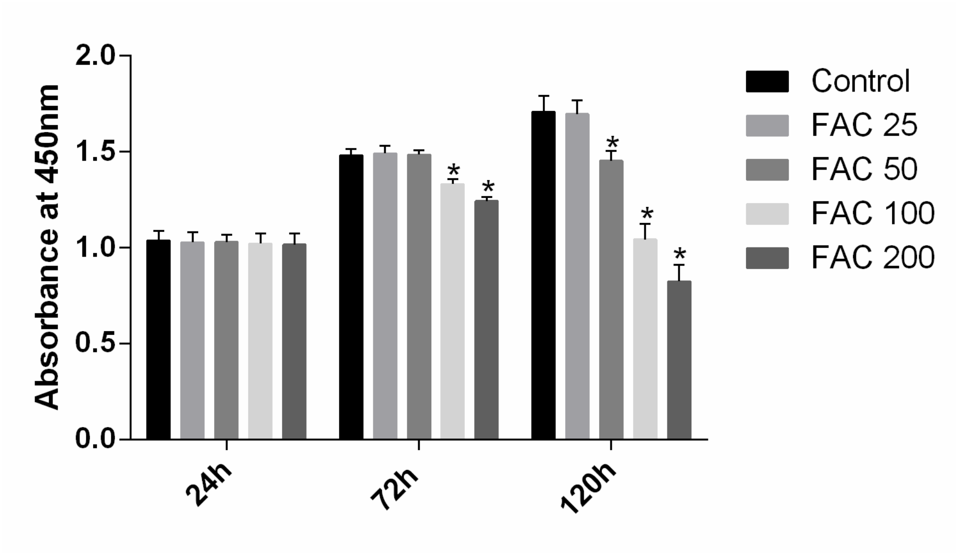

Figure 2 Effect of iron-overload on the intracellular LIP in osteoblasts.

Labile iron pool	Control	FAC 25	FAC 50	FAC 100	FAC 200
Group1					
No:	131226.003	131226.004	131226.005	131226.006	131226.007
MFI(mean fluorescent intensity)	172.77	153.82	130.58	74.88	66.56
Group2					
No:	140114.001	140114.002	140114.003	140114.004	140114.005
MFI(mean fluorescent intensity)	147.92	117.21	108.56	66.75	48.41
Group3					
No:	140116.001	140116.002	140116.003	140116.004	140116.005
MFI(mean fluorescent intensity)	175.87	124.42	98.45	88.52	75.32

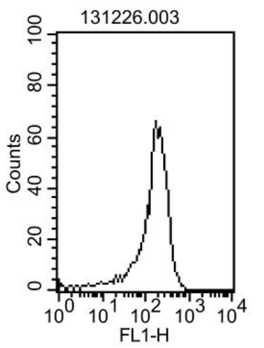

Marker	% Gated	Mean	Geo Mean
All	100.00	172.77	146.31

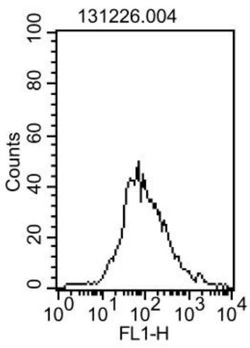

Marker	% Gated	Mean	Geo Mean
All	100.00	153.82	87.66

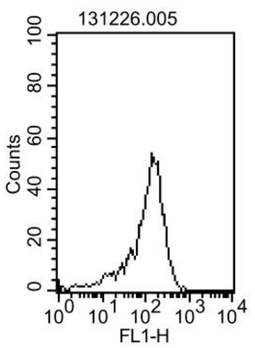

Marker	% Gated	Mean	Geo Mean
All	100.00	130.58	103.03

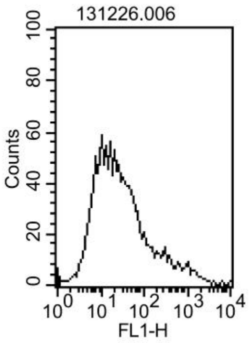

Marker	% Gated	Mean	Geo Mean
All	100.00	74.88	25.68

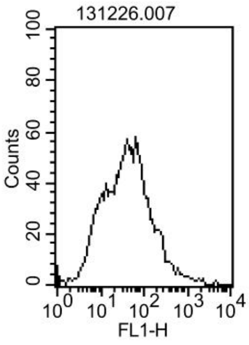

Marker	% Gated	Mean	Geo Mean
All	100.00	66.56	35.45

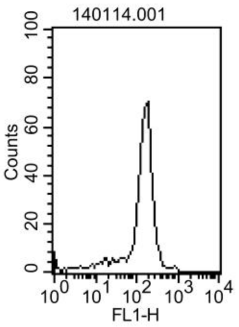

Marker	% Gated	Mean	Geo Mean
All	100.00	147.92	128.50

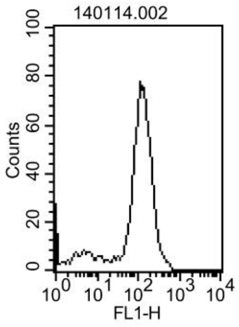

Marker	% Gated	Mean	Geo Mean
All	100.00	117.21	89.29

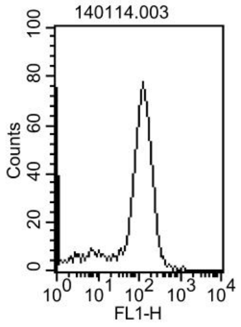

Marker	% Gated	Mean	Geo Mean
All	100.00	108.56	78.20

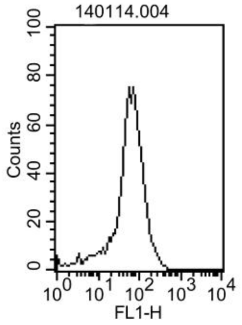

Marker	% Gated	Mean	Geo Mean
All	100.00	66.75	54.35

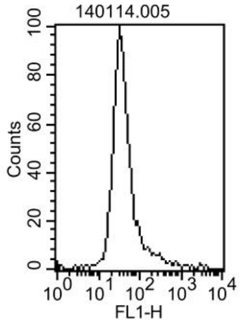

Marker	% Gated	Mean	Geo Mean
All	100.00	48.41	37.21

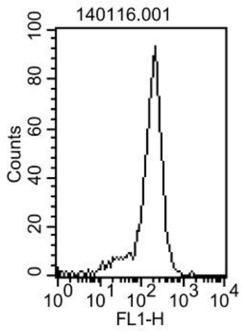

Marker	% Gated	Mean	Geo Mean
All	100.00	175.87	150.77

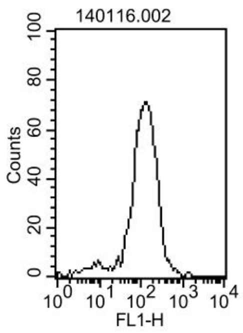

Marker	% Gated	Mean	Geo Mean
All	100.00	124.42	103.60

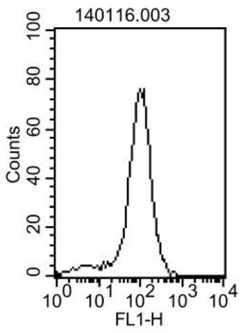

Marker	% Gated	Mean	Geo Mean
All	100.00	98.45	81.17

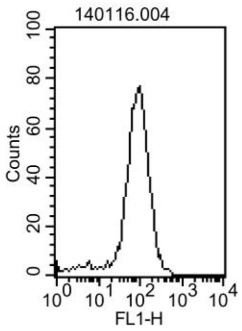

Marker	% Gated	Mean	Geo Mean
All	100.00	88.52	73.27

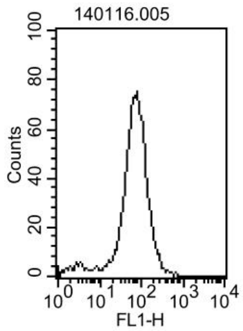

Marker	% Gated	Mean	Geo Mean
All	100.00	75.32	62.66

Control

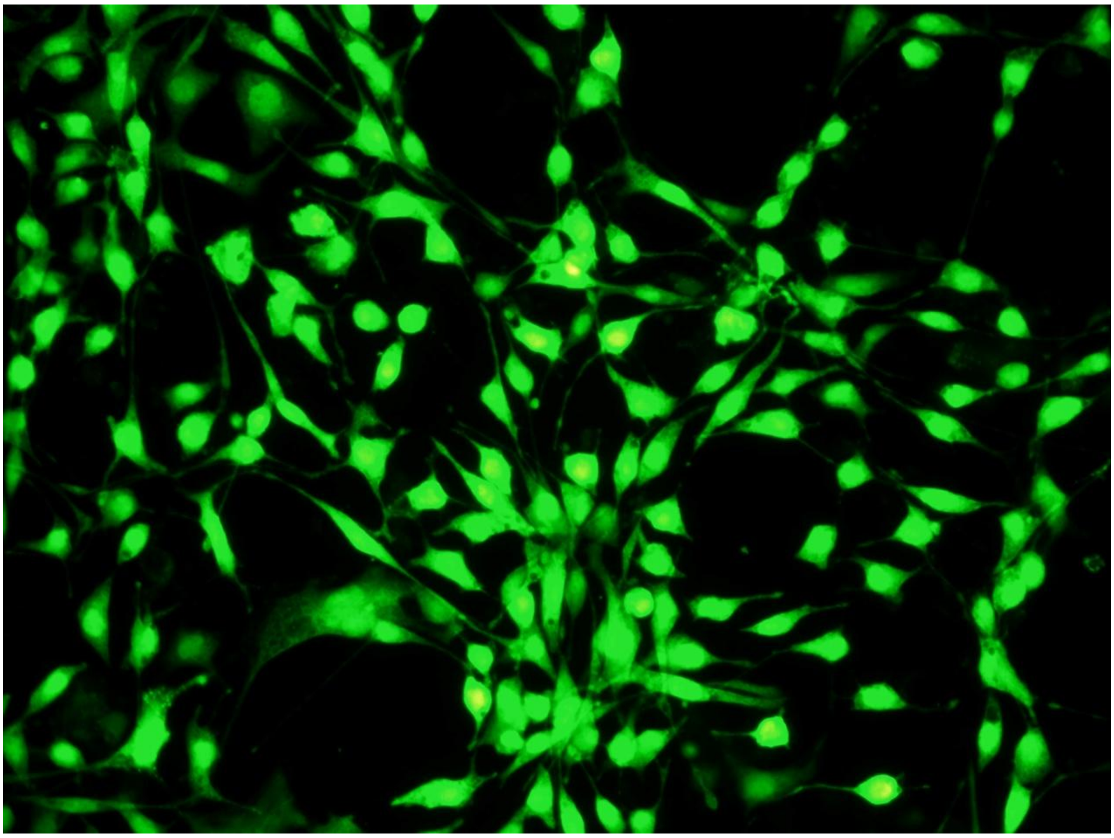

FAC 25

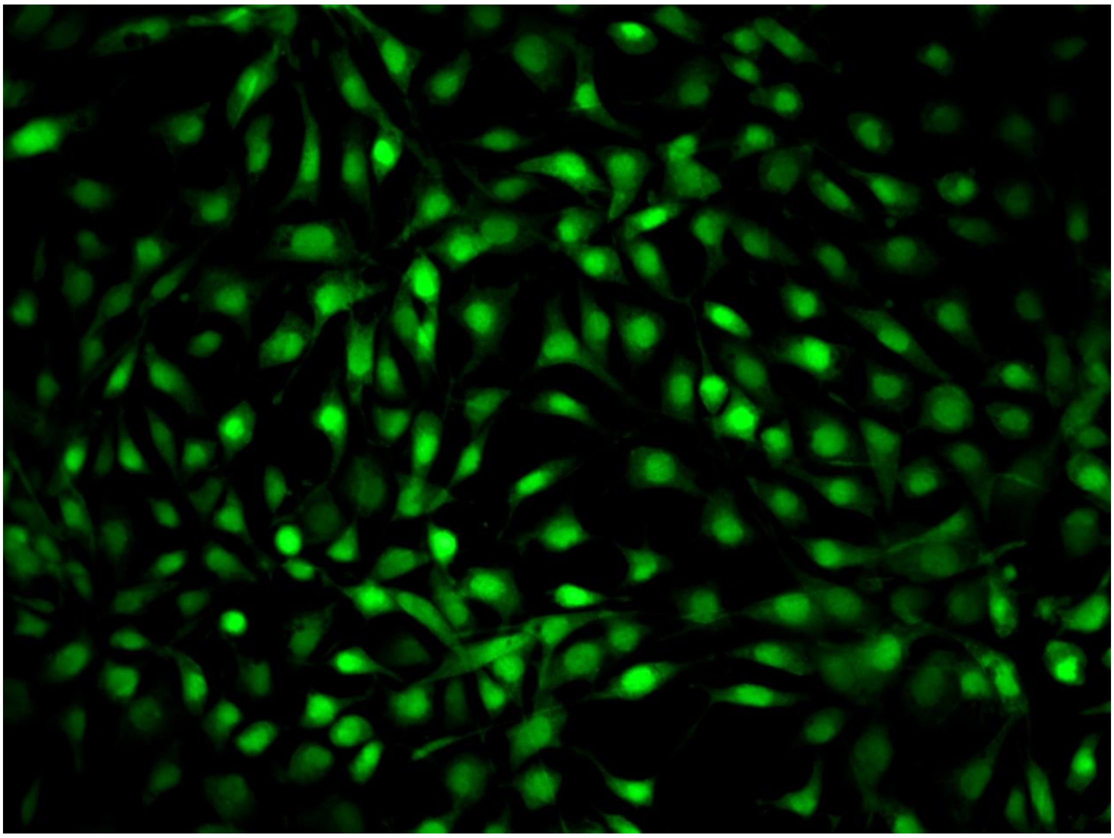

FAC 50

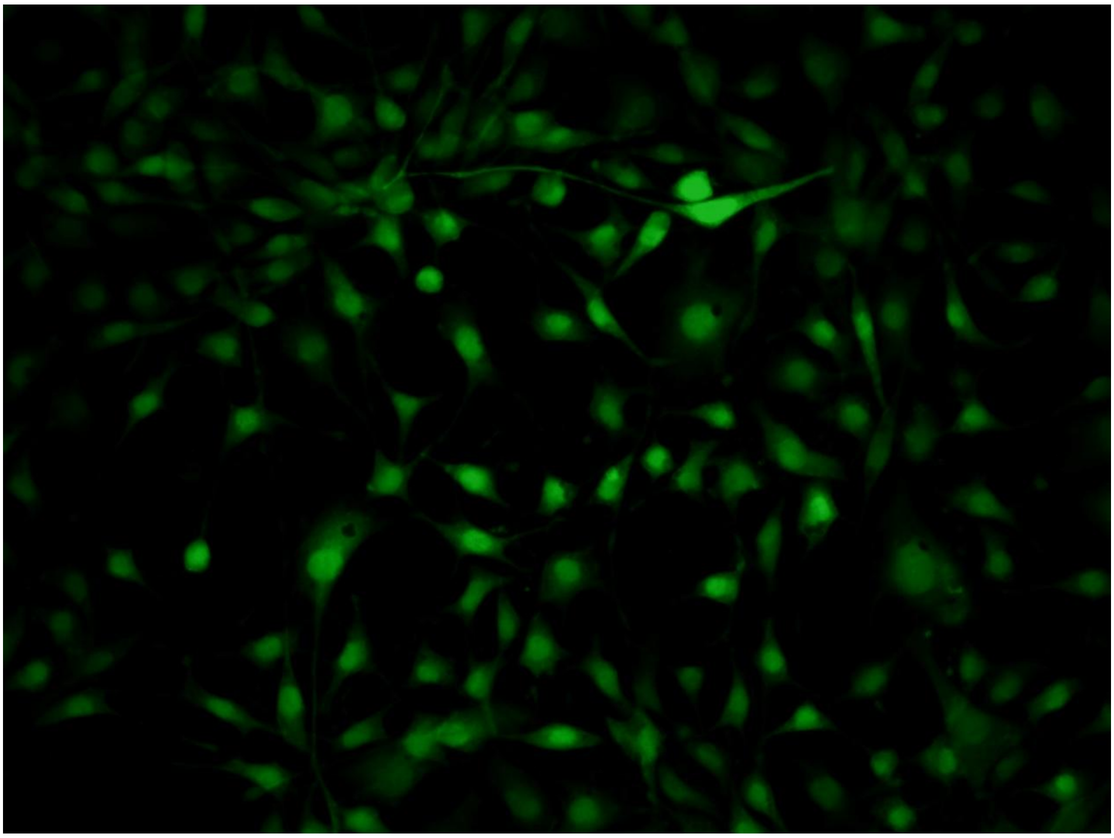

FAC 100

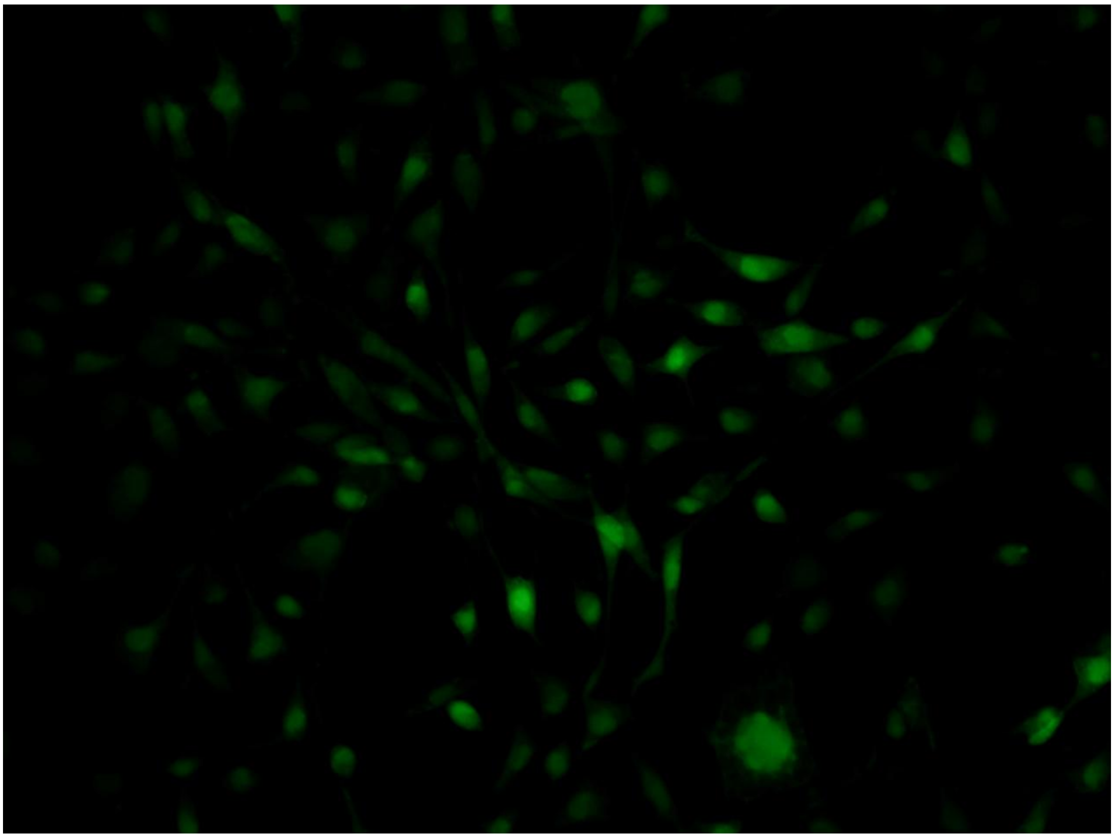

FAC 200

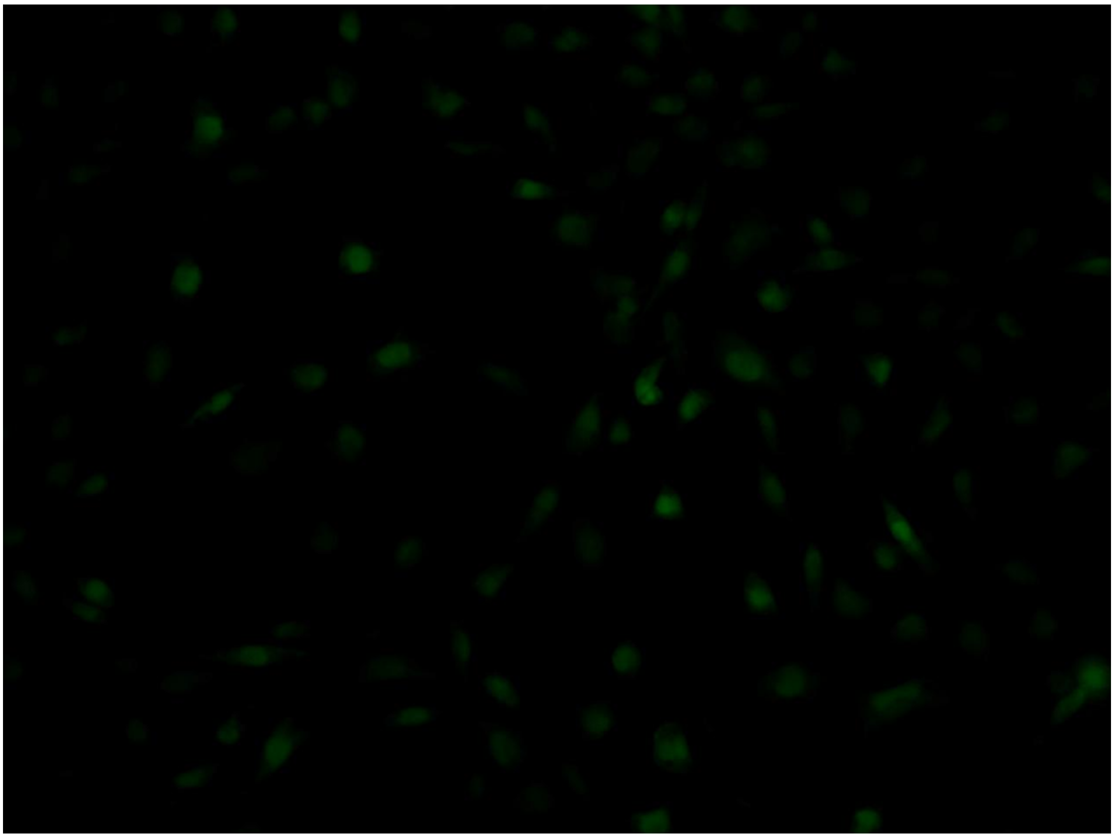

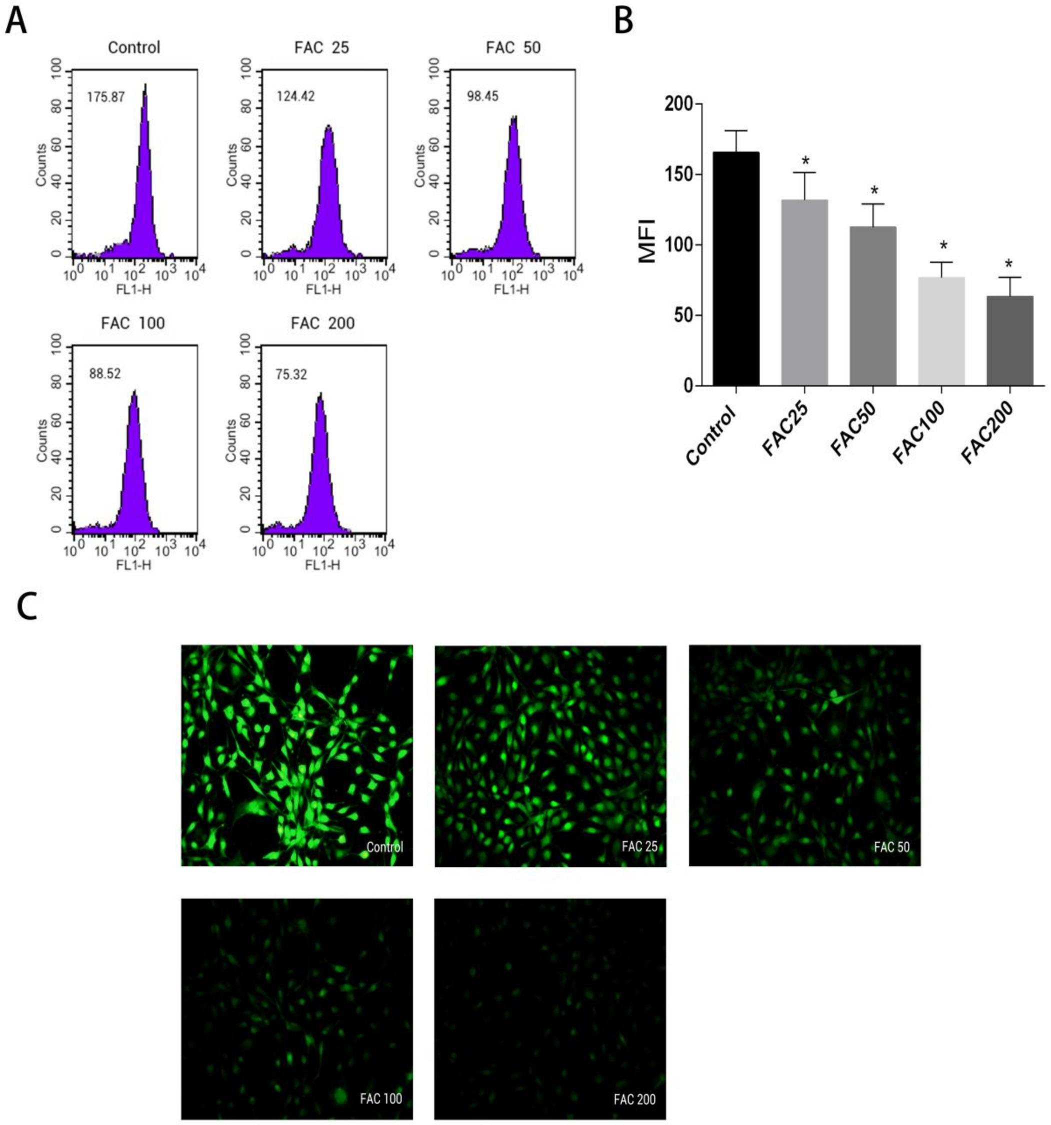
A
Control
175.87
FAC 25
124.42
FAC 50
98.45
FAC 100
88.52
FAC 200
75.32
Counts
FL1-H
B
MFI
Control
FAC25
FAC50
FAC100
FAC200
C
Control
FAC 25
FAC 50
FAC 100
FAC 200

Figure 3 Iron induced ROS generation and upregulation of Nox4 in osteoblasts.

Reactive Oxygen Species(ROS)	Control	FAC 25	FAC 50	FAC 100	FAC 200
Group1					
No:	131007.001	131007.002	131007.003	131007.004	131007.005
MFI(mean fluorescent intensity)	4.84	11.72	25.52	59.07	64.93
Group2					
No:	131121.001	131121.002	131121.003	131121.004	131121.005
MFI(mean fluorescent intensity)	8.37	11.89	24.35	44.41	73.59
Group3					
No:	131122.001	131122.002	131122.003	131122.004	131122.005
MFI(mean fluorescent intensity)	7.13	11.59	27.26	48.41	68.98

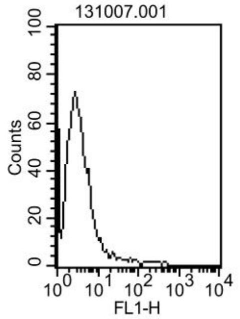

Marker	% Gated	Mean	Geo Mean
All	100.00	4.84	3.32

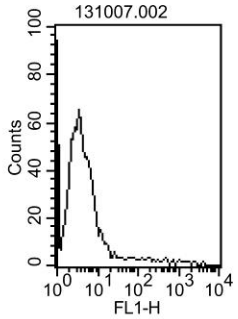

Marker	% Gated	Mean	Geo Mean
All	100.00	11.72	4.24

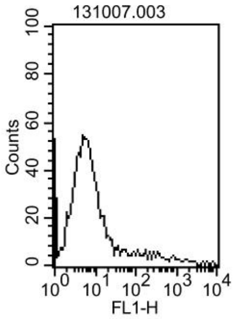

Marker	% Gated	Mean	Geo Mean
All	100.00	25.52	6.90

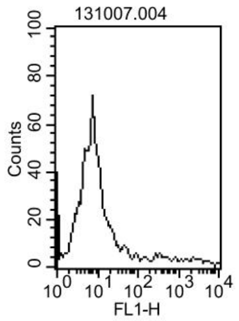

Marker	% Gated	Mean	Geo Mean
All	100.00	59.07	9.38

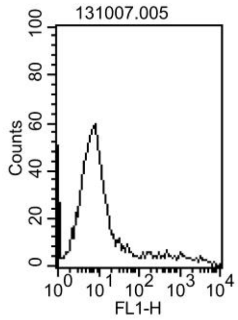

Marker	% Gated	Mean	Geo Mean
All	100.00	64.93	10.17

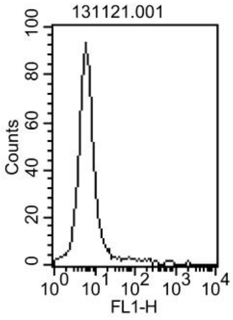

Marker	% Gated	Mean	Geo Mean
All	100.00	8.37	6.37

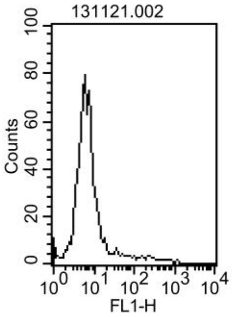

Marker	% Gated	Mean	Geo Mean
All	100.00	11.89	6.88

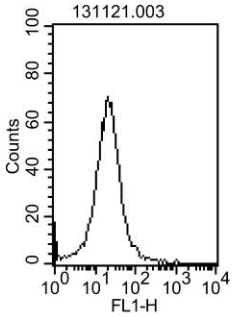

Marker	% Gated	Mean	Geo Mean
All	100.00	24.35	19.33

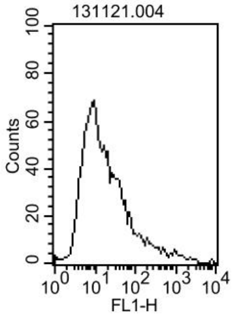

Marker	% Gated	Mean	Geo Mean
All	100.00	44.41	16.02

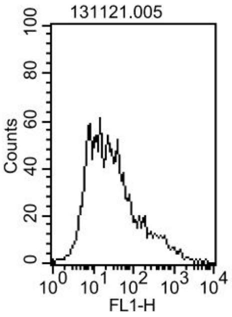

Marker	% Gated	Mean	Geo Mean
All	100.00	73.59	26.61

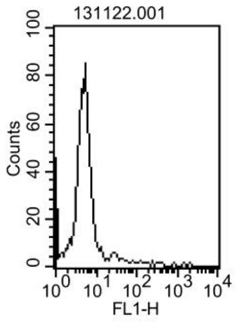

Marker	% Gated	Mean	Geo Mean
All	100.00	7.13	4.97

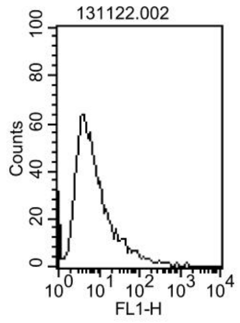

Marker	% Gated	Mean	Geo Mean
All	100.00	11.59	6.60

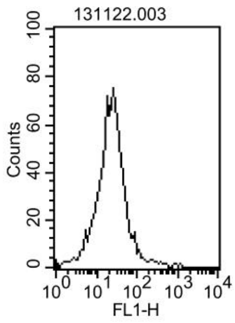

Marker	% Gated	Mean	Geo Mean
All	100.00	27.26	21.21

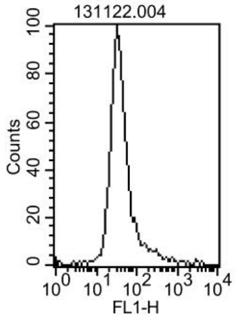

Marker	% Gated	Mean	Geo Mean
All	100.00	48.41	37.21

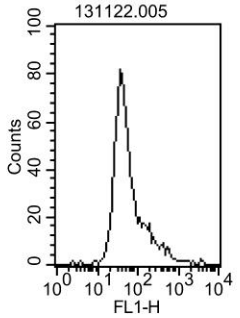

Marker	% Gated	Mean	Geo Mean
All	100.00	68.98	48.76

WB

Beta-actin Control FAC 25 FAC 50 FAC 100 FAC200

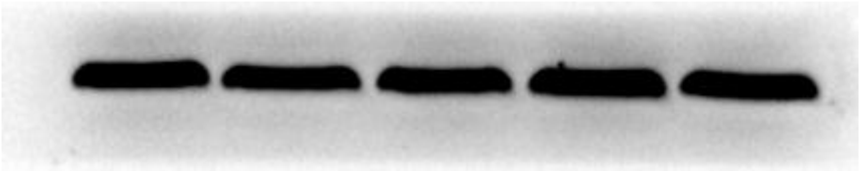

Nox4

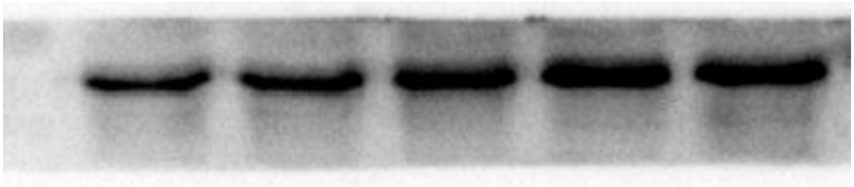

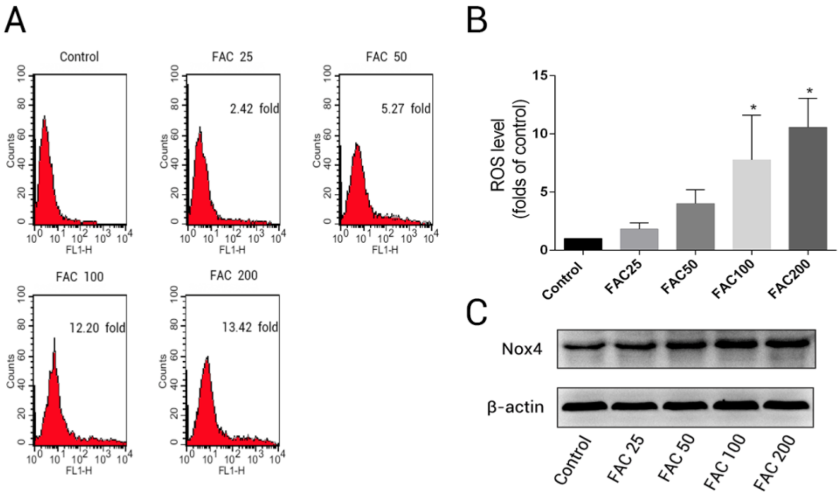
A
Control
FAC 25
2.42 fold
FAC 50
5.27 fold
FAC 100
12.20 fold
FAC 200
13.42 fold
Counts
FL1-H
B
ROS level (folds of control)
Control
FAC25
FAC50
FAC100
FAC200
C
Nox4
β-actin
Control
FAC 25
FAC 50
FAC 100
FAC 200

Figure 4 Iron induced apoptosis in osteoblasts.

Apoptosis rate(%)	Control	FAC 25	FAC 50	FAC 100	FAC 200
Group1					
No:	131121.001	131121.002	131121.003	131121.004	131121.005
Early apoptosis	1.04	1.55	3.72	11.32	17.9
Late apoptosis	3.31	5.43	10	13.44	36.02
Group2					
No:	131202.001	131202.002	131202.003	131202.004	131202.005
Early apoptosis	0.91	4.44	11.36	14	27.69
Late apoptosis	3.06	5.22	5.36	8.4	35.89
Group3					
No:	131104.001	131104.002	131104.003	131104.004	131104.005
Early apoptosis	0.65	0.94	0.93	1.29	15.18
Late apoptosis	3.46	6.59	14.4	21.3	37.48

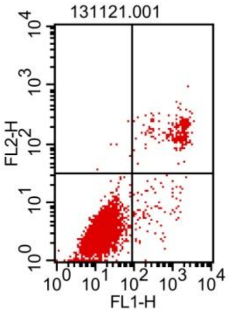

Quad	% Gated
UL	0.07
UR	3.31
LL	95.59
LR	1.04

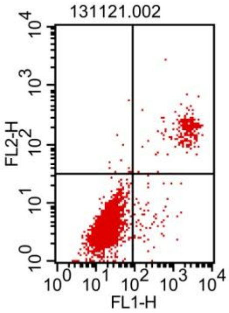

Quad	% Gated
UL	0.21
UR	5.43
LL	92.81
LR	1.55

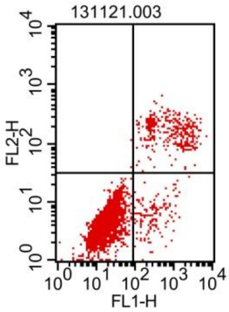

Quad	% Gated
UL	0.08
UR	10.00
LL	86.20
LR	3.72

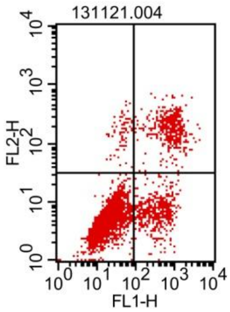

Quad	% Gated
UL	1.88
UR	13.44
LL	73.36
LR	11.32

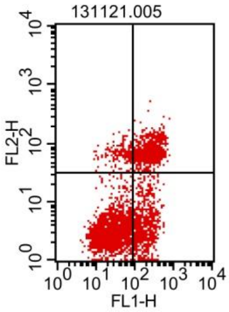

Quad	% Gated
UL	4.88
UR	36.02
LL	41.20
LR	17.90

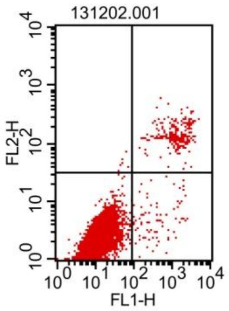

Quad	% Gated
UL	0.07
UR	3.06
LL	95.97
LR	0.91

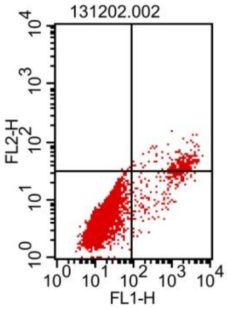

Quad	% Gated
UL	0.14
UR	5.22
LL	90.20
LR	4.44

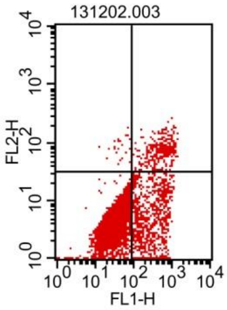

Quad	% Gated
UL	0.36
UR	5.36
LL	82.92
LR	11.36

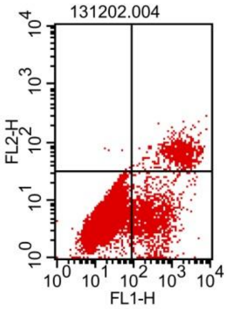

Quad	% Gated
UL	0.08
UR	8.40
LL	77.53
LR	14.00

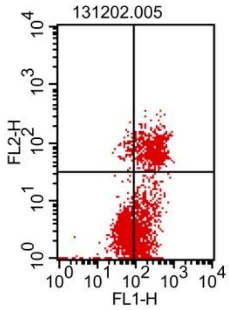

Quad	% Gated
UL	2.02
UR	35.89
LL	34.40
LR	27.69

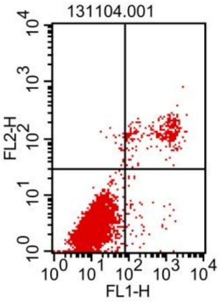

Quad	% Gated
UL	0.29
UR	3.46
LL	95.60
LR	0.65

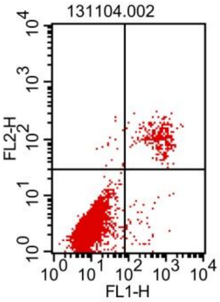

Quad	% Gated
UL	0.44
UR	6.59
LL	92.03
LR	0.94

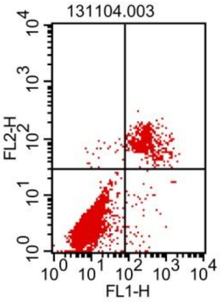

Quad	% Gated
UL	0.56
UR	14.40
LL	84.11
LR	0.93

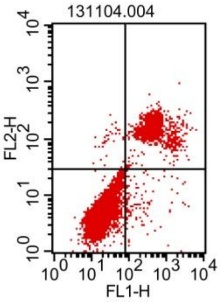

Quad	% Gated
UL	0.49
UR	21.30
LL	76.92
LR	1.29

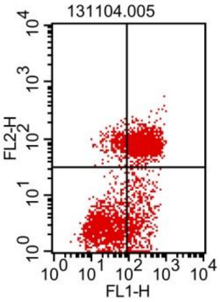

Quad	% Gated
UL	8.30
UR	37.48
LL	39.03
LR	15.18

Control

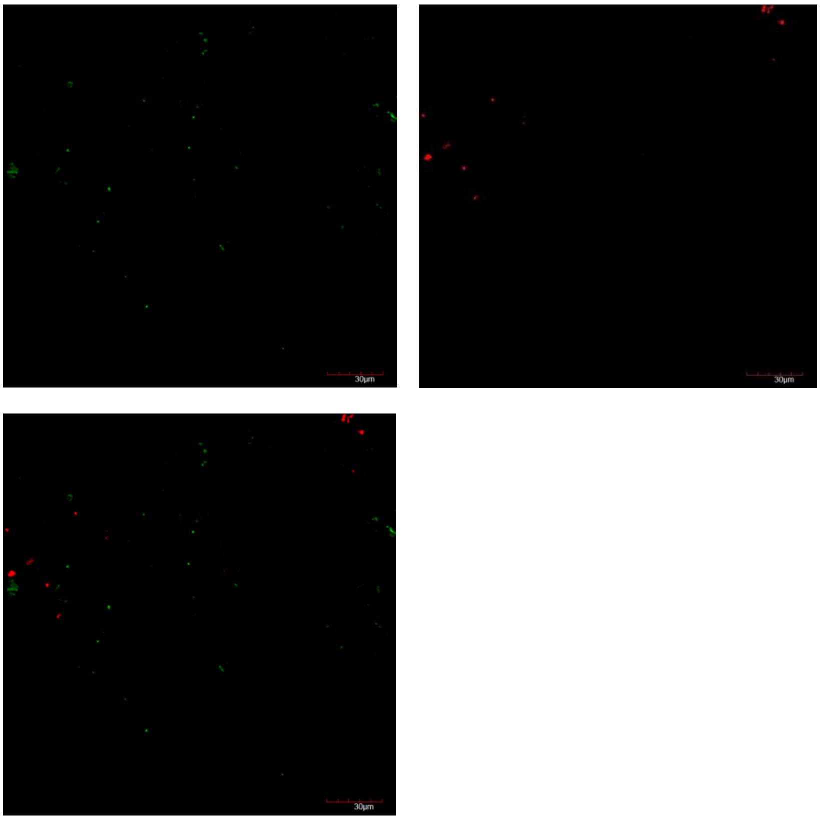

FAC 200

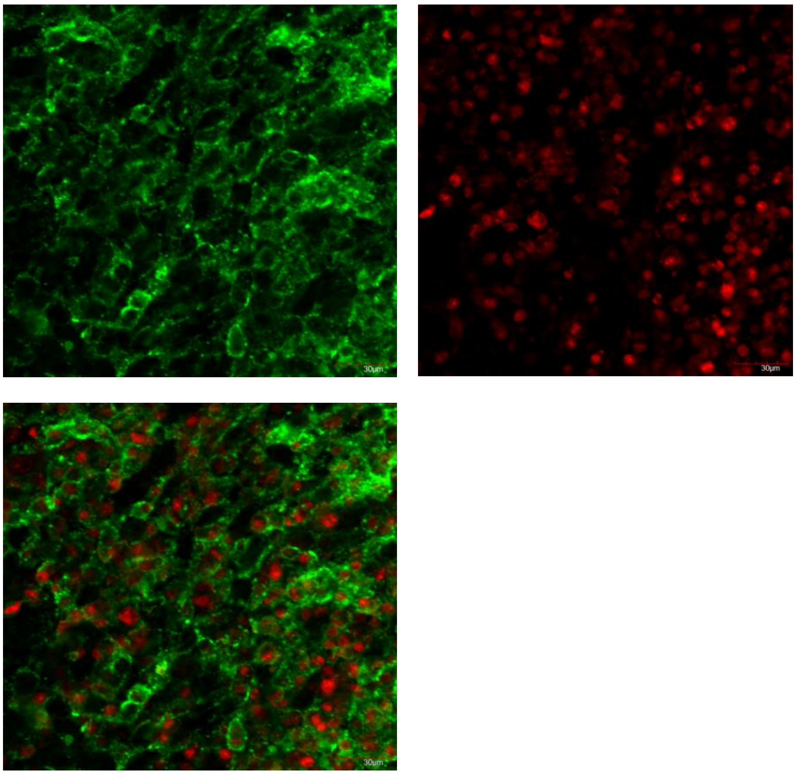

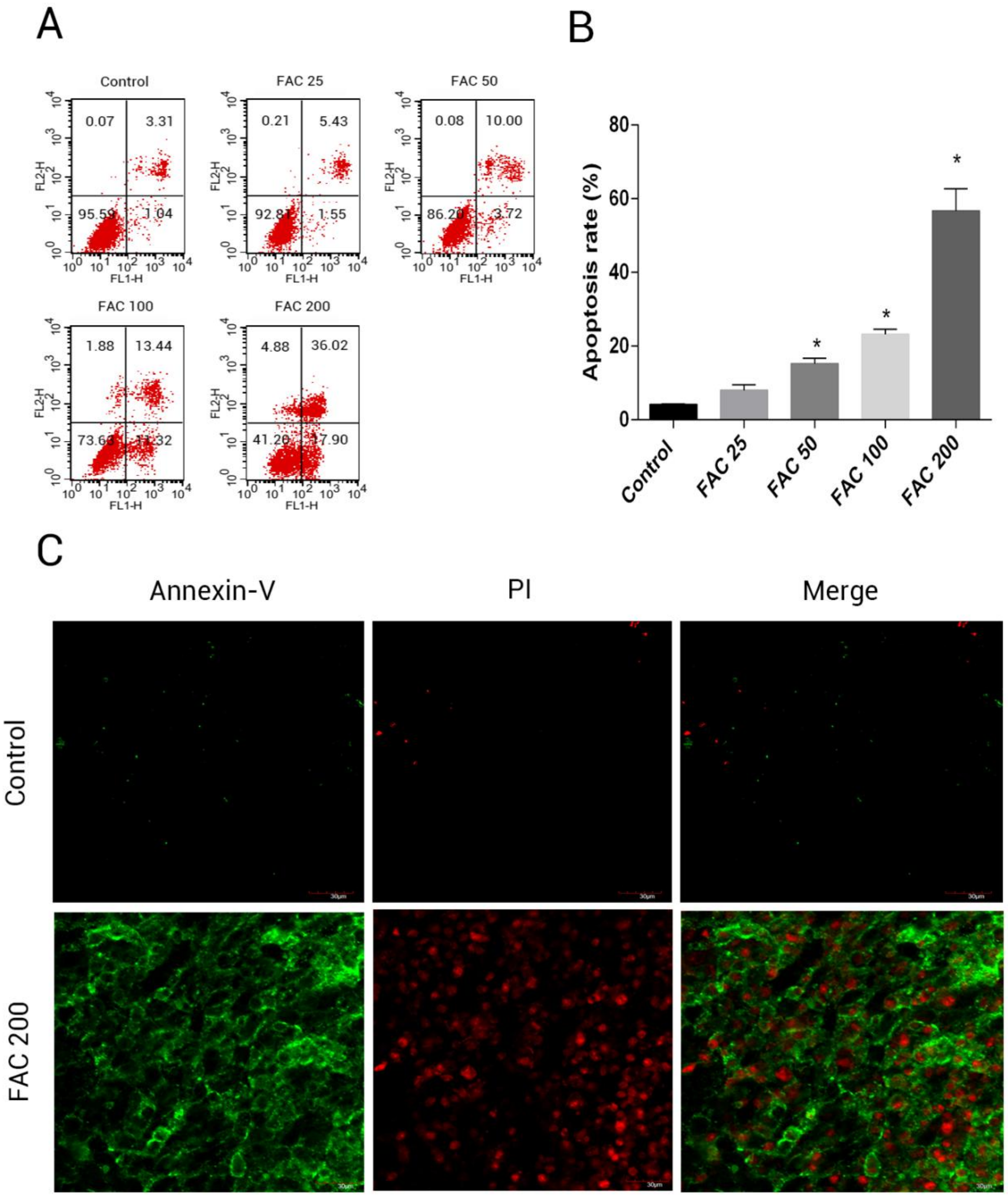
A
B
C
Control
FAC 25
FAC 50
FAC 100
FAC 200
FL1-H
FL2-H
0.07
3.31
95.59
1.04
0.21
5.43
92.81
1.55
0.08
10.00
86.20
3.72
1.88
13.44
73.63
11.32
4.88
36.02
41.20
17.90
Apoptosis rate (%)
Annexin-V
PI
Merge

Figure 5 The morphological changes of apoptosis in osteoblasts FAC.

Control

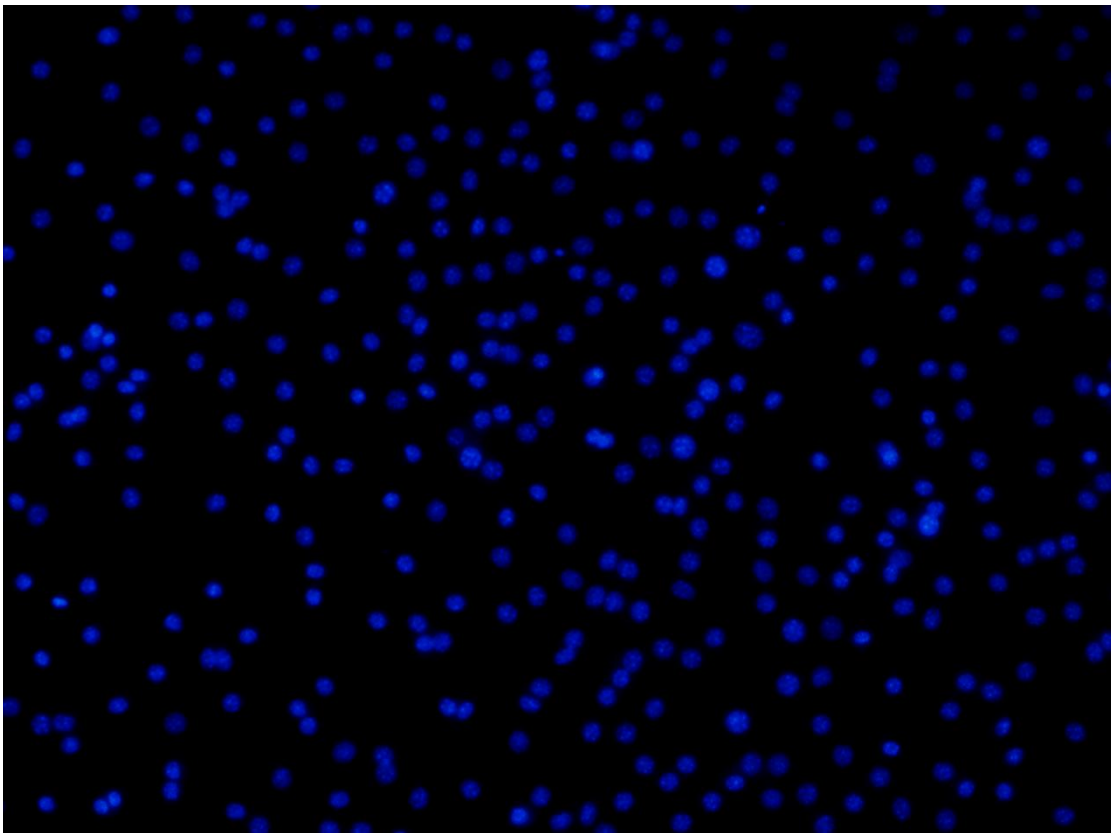

FAC 200

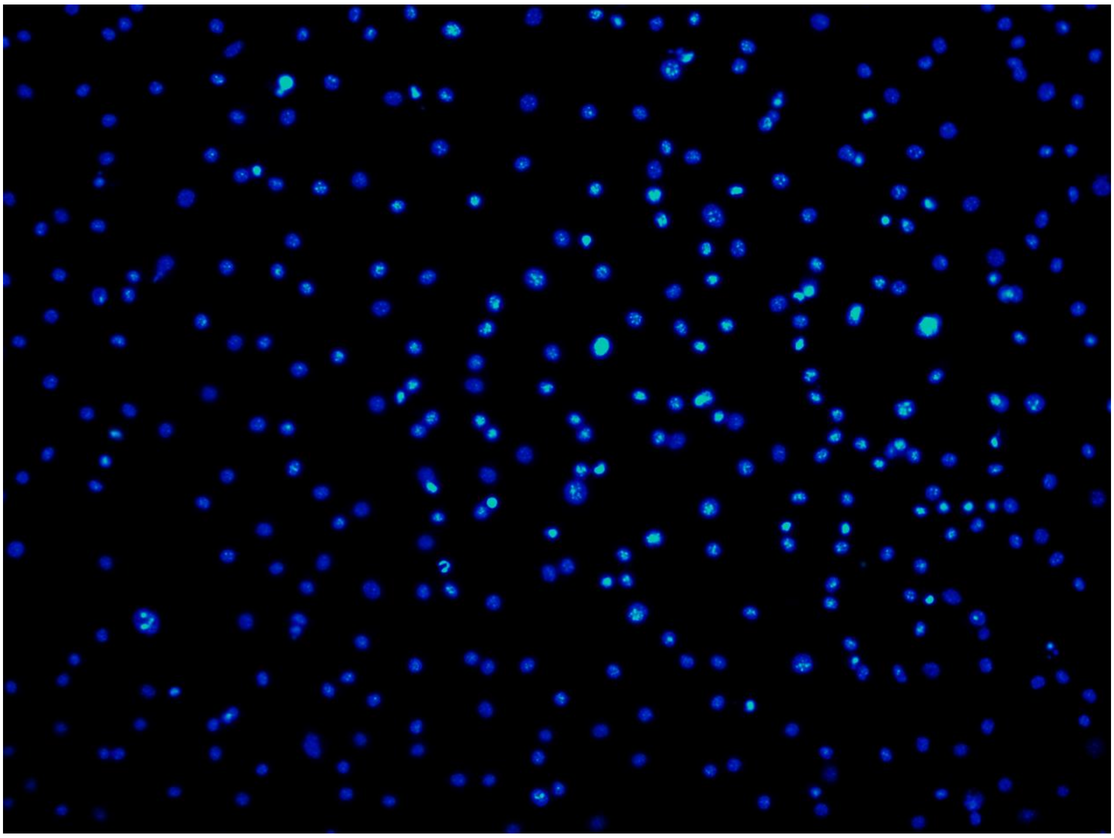

Control

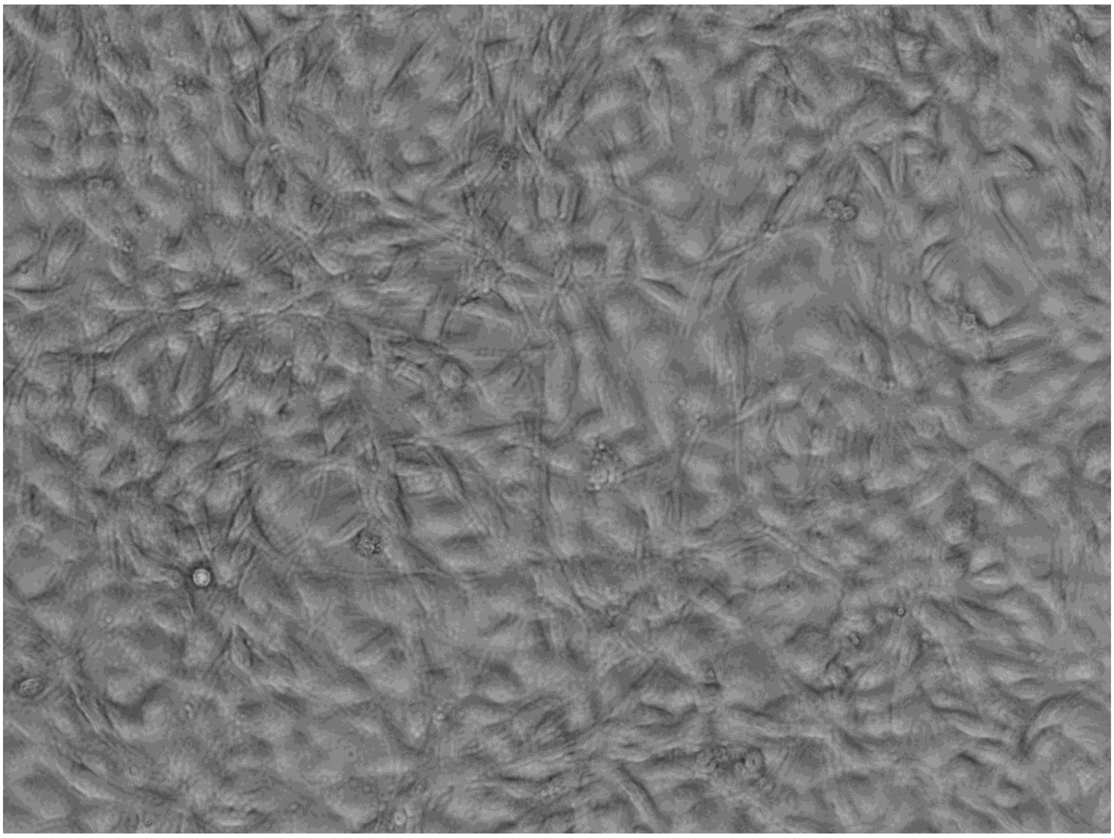

FAC 200

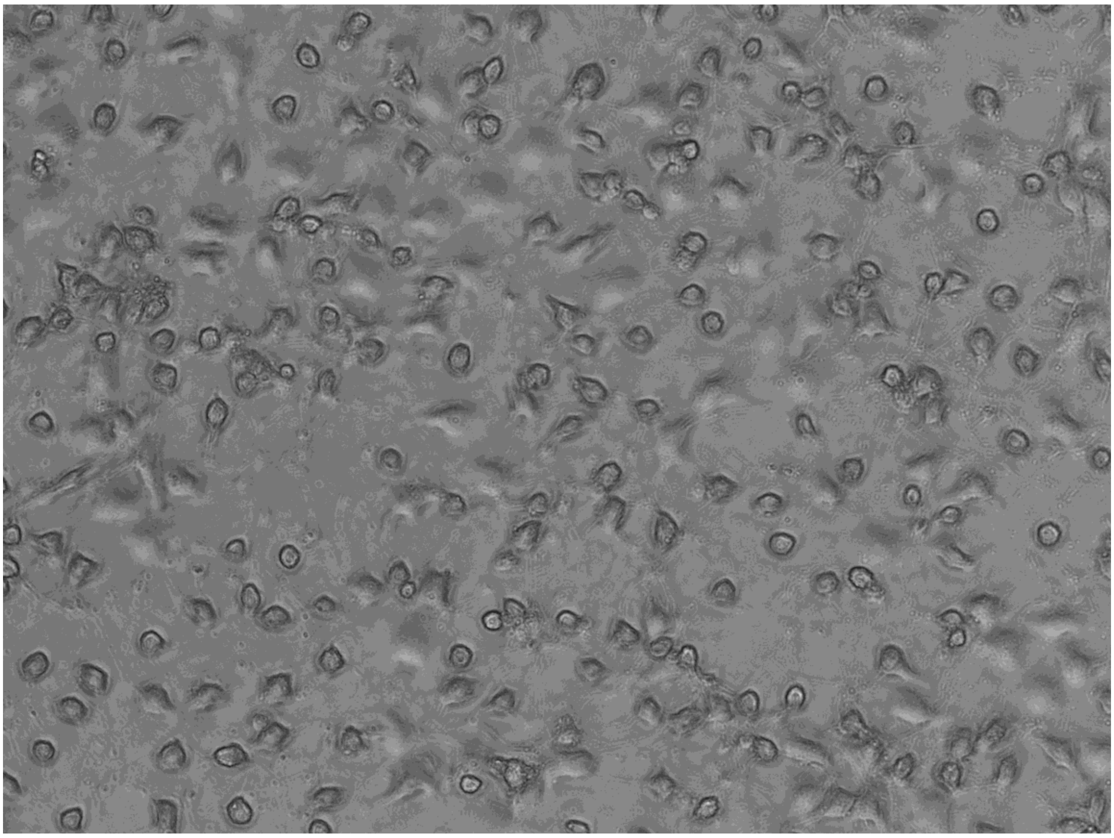

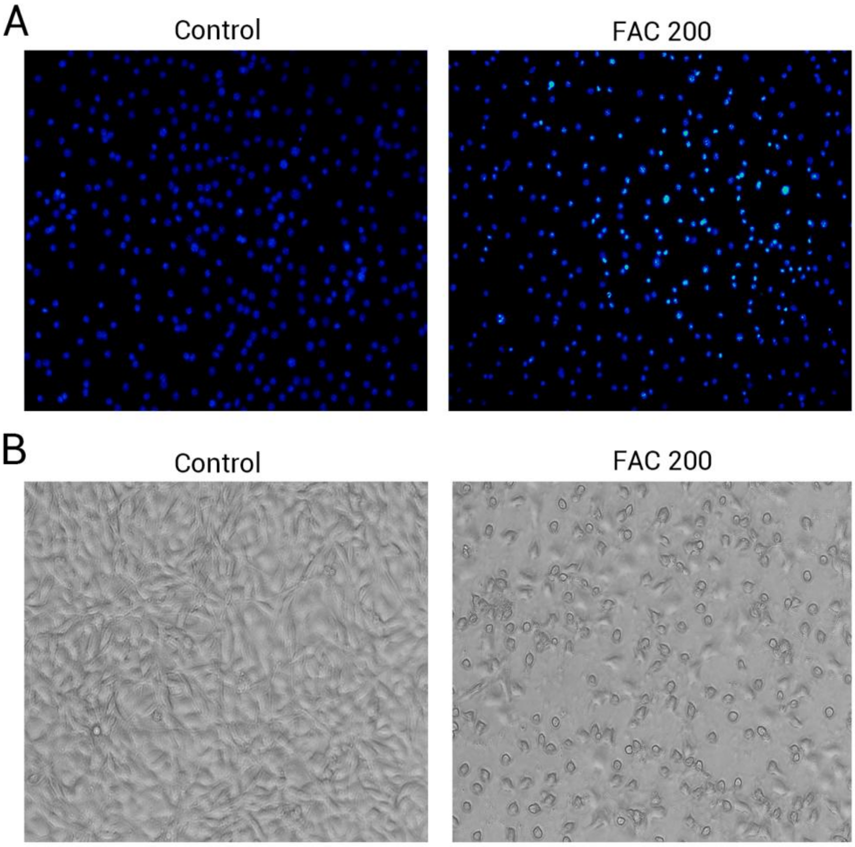

Figure 6 The expression of apoptosis-related proteins in osteoblasts.

GAPDH **Control** **FAC 25** **FAC 50** **FAC 100** **FAC200**

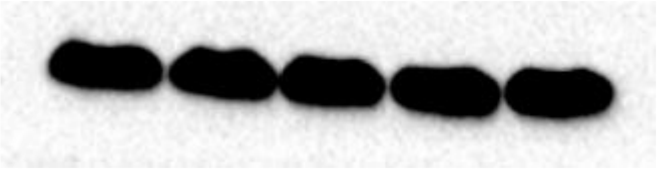

Cleaved caspase3

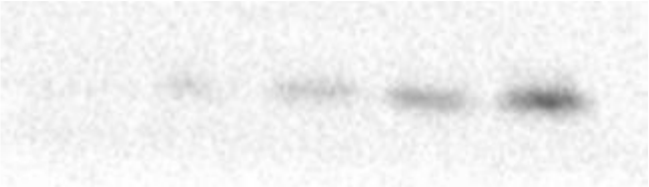

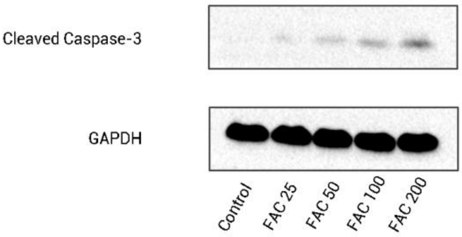

GAPDH **Control FAC 25 FAC 50 FAC 100 FAC200**

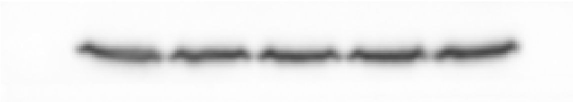

Bcl-2

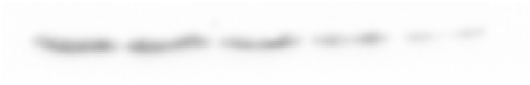

Bax

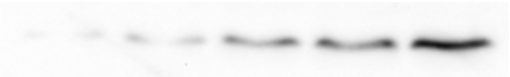

Cytochrome c

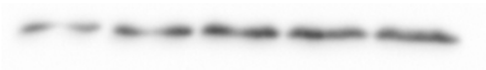

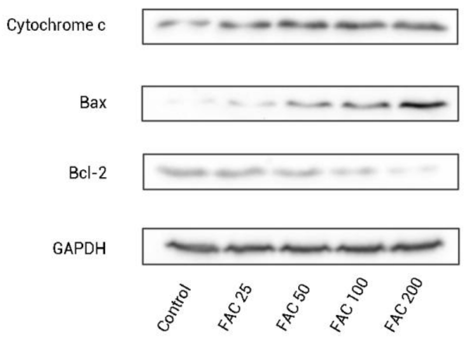

Figure 7 Iron-induced decrease in MMP of osteoblasts.

Mitochondrial membrane potential (MMP)	Control	FAC 25	FAC 50	FAC 100	FAC 200
Group1					
No:	131017.001	131017.002	131017.003	131017.004	131017.005
Red fluorescent intensity	91.44	88.92	70.22	63.14	41.41
Green fluorescent intensity	8.55	11.07	29.79	36.8	58.54
Group2					
No:	131025.001	131025.002	131025.003	131025.004	131025.005
Red fluorescent intensity	94.78	82.3	74.39	66.99	42.75
Green fluorescent intensity	5	17.52	25.59	32.95	57.23
Group3					
No:	131217.001	131217.002	131217.003	131217.004	131217.005
Red fluorescent intensity	90.48	88.47	76.67	56.99	31.6
Green fluorescent intensity	9.51	11.48	23.32	42.84	68.35

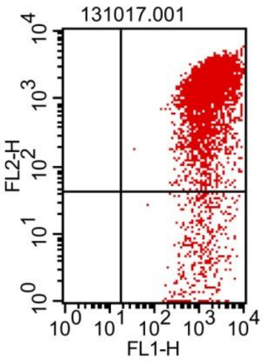

Quad	% Gated
UL	0.00
UR	91.44
LL	0.01
LR	8.55

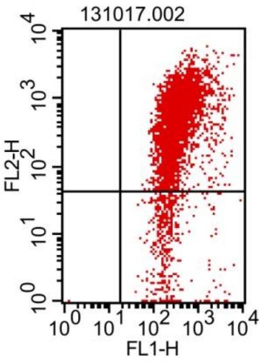

Quad	% Gated
UL	0.00
UR	88.92
LL	0.01
LR	11.07

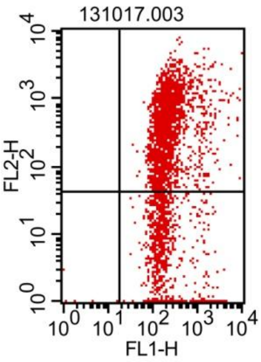

Quad	% Gated
UL	0.00
UR	70.02
LL	0.19
LR	29.79

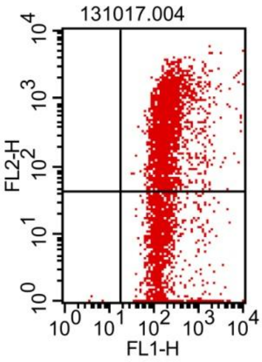

Quad	% Gated
UL	0.00
UR	63.14
LL	0.06
LR	36.80

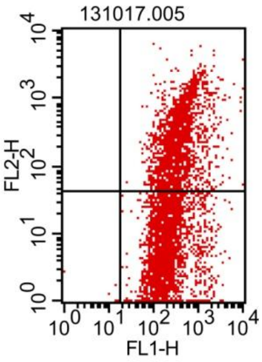

Quad	% Gated
UL	0.00
UR	41.41
LL	0.06
LR	58.54

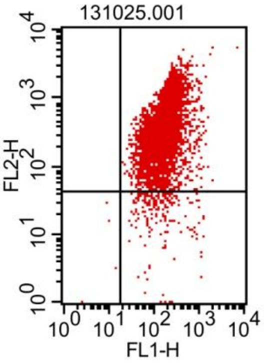

Quad	% Gated
UL	0.03
UR	94.78
LL	0.19
LR	5.00

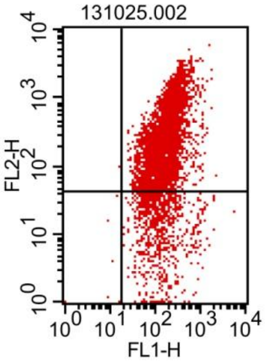

Quad	% Gated
UL	0.02
UR	82.30
LL	0.15
LR	17.52

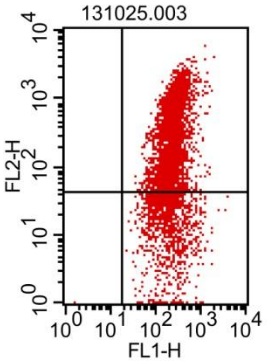

Quad	% Gated
UL	0.00
UR	74.39
LL	0.02
LR	25.59

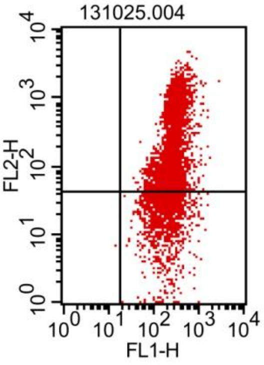

Quad	% Gated
UL	0.00
UR	66.99
LL	0.05
LR	32.95

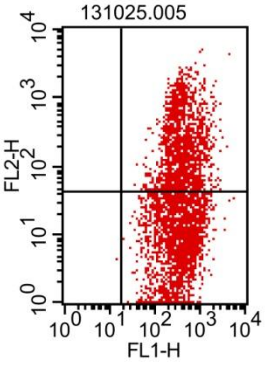

Quad	% Gated
UL	0.00
UR	42.75
LL	0.03
LR	57.23

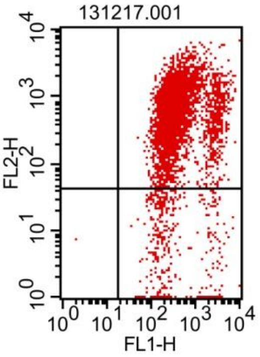

Quad	% Gated
UL	0.00
UR	90.48
LL	0.01
LR	9.51

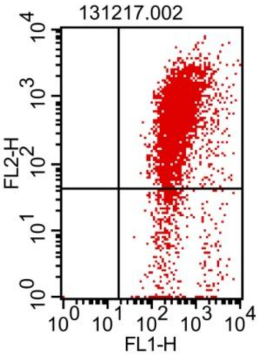

Quad	% Gated
UL	0.00
UR	88.47
LL	0.05
LR	11.48

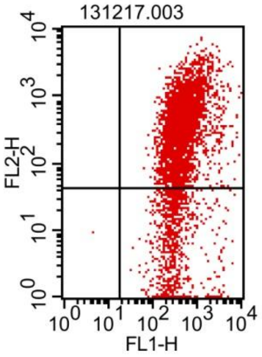

Quad	% Gated
UL	0.00
UR	76.67
LL	0.01
LR	23.32

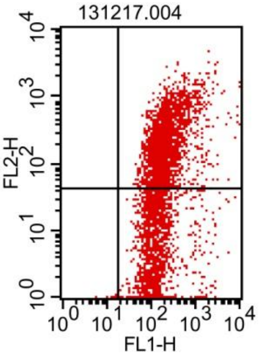

Quad	% Gated
UL	0.00
UR	56.99
LL	0.17
LR	42.84

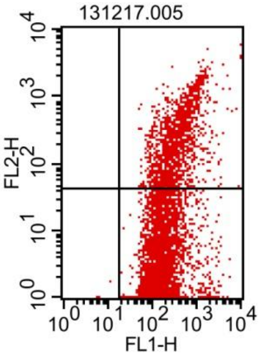

Quad	% Gated
UL	0.00
UR	31.60
LL	0.04
LR	68.35

Control

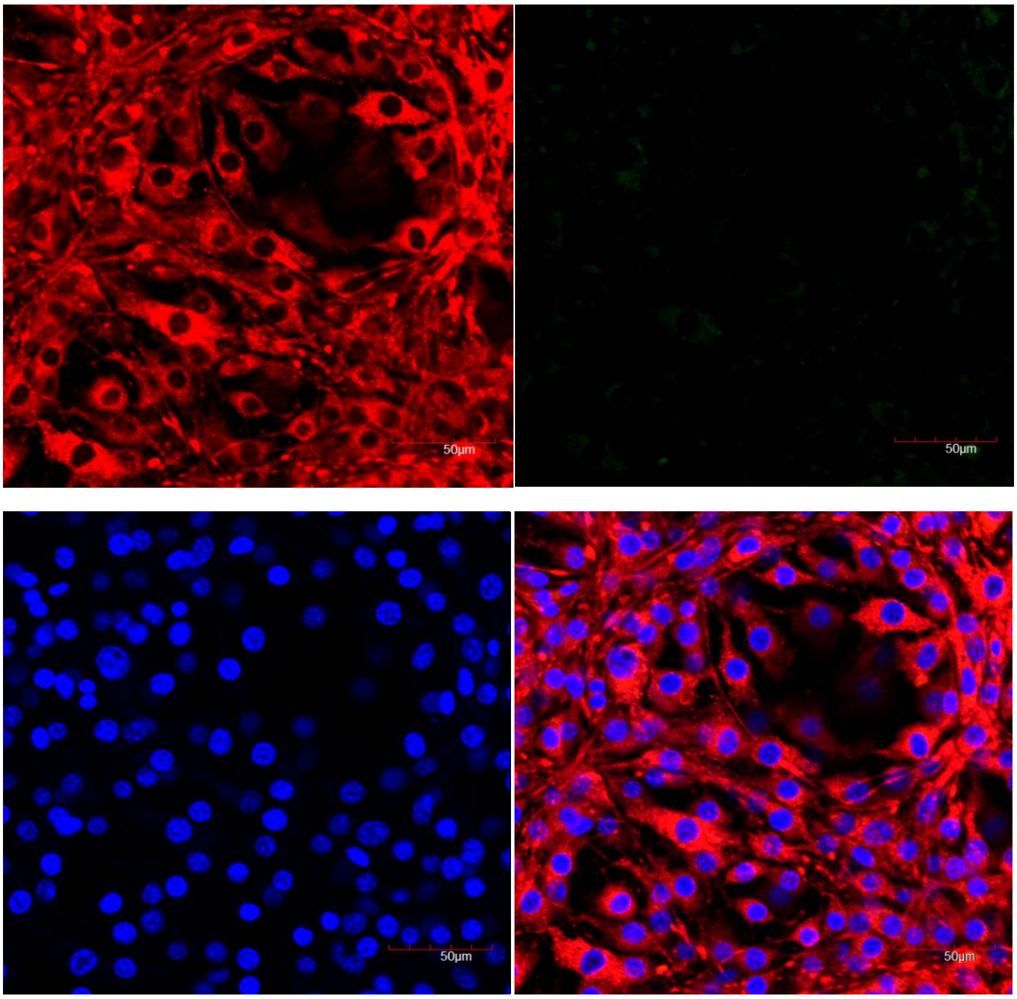

FAC 25

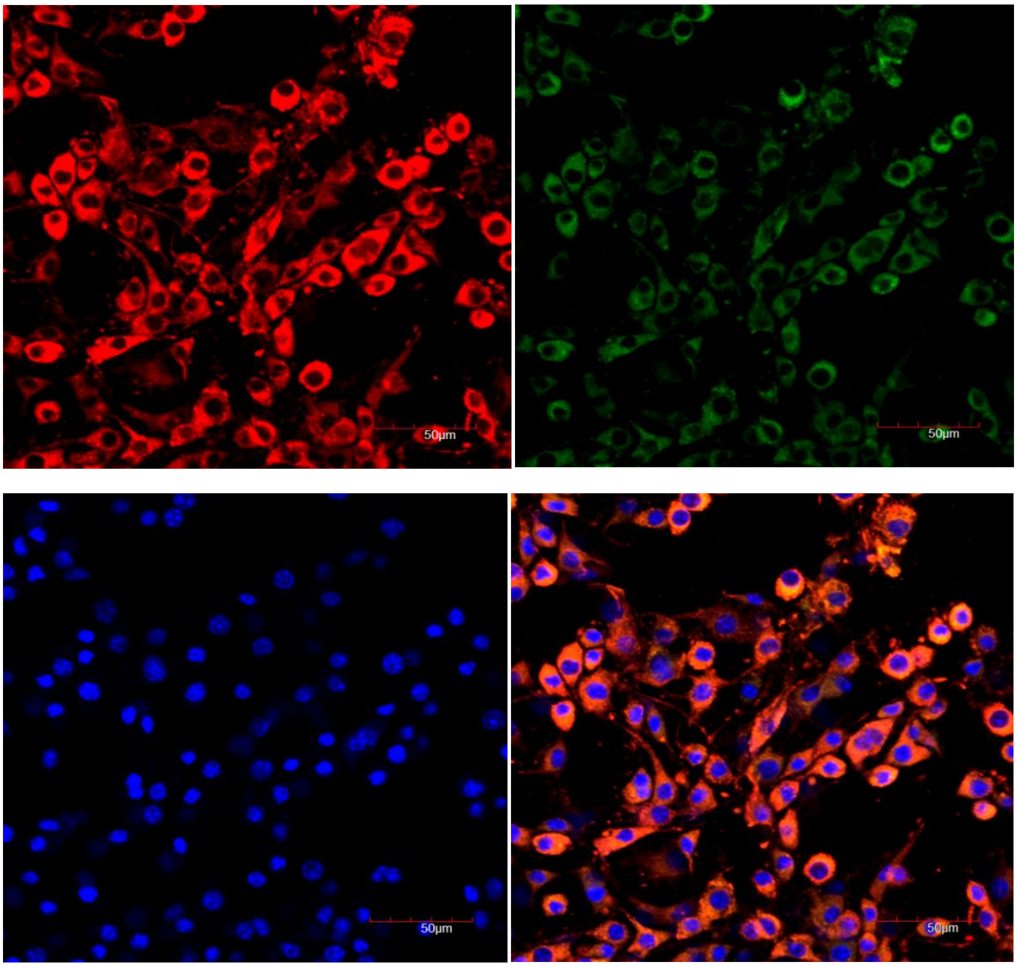

FAC 50

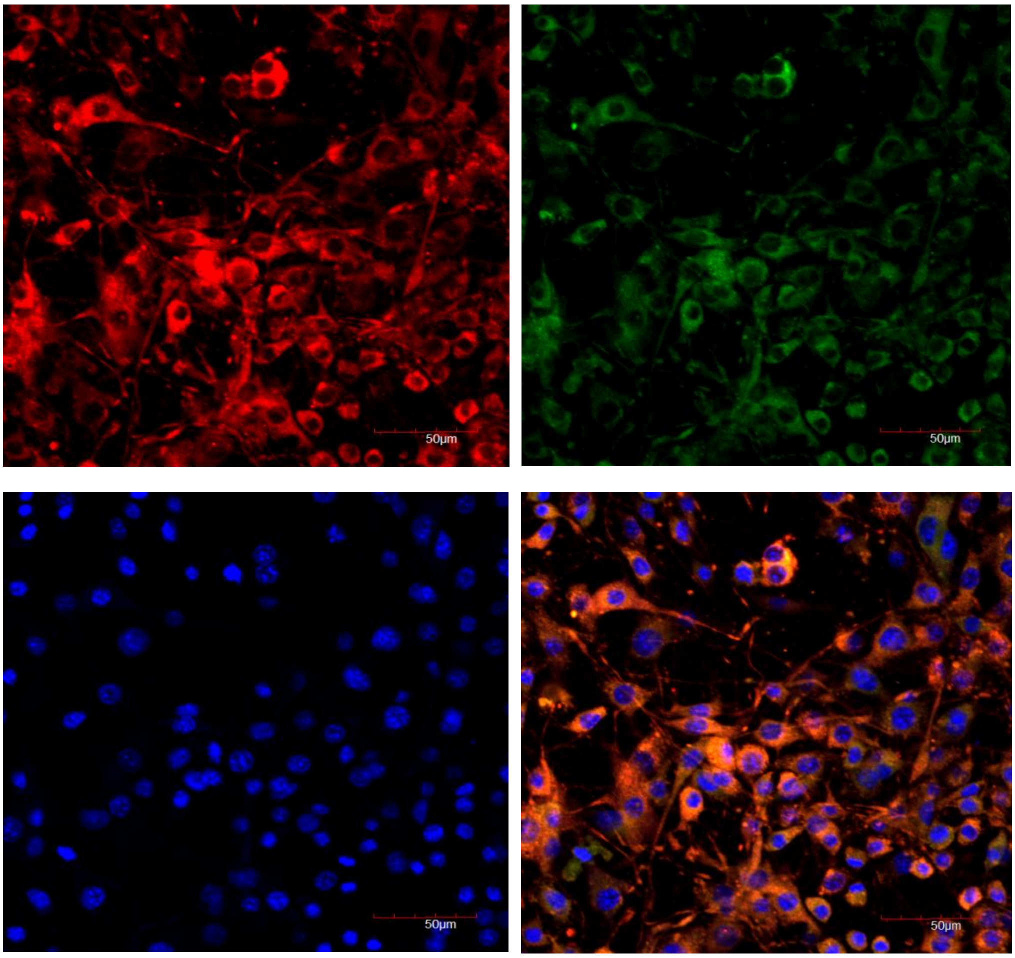

FAC 100

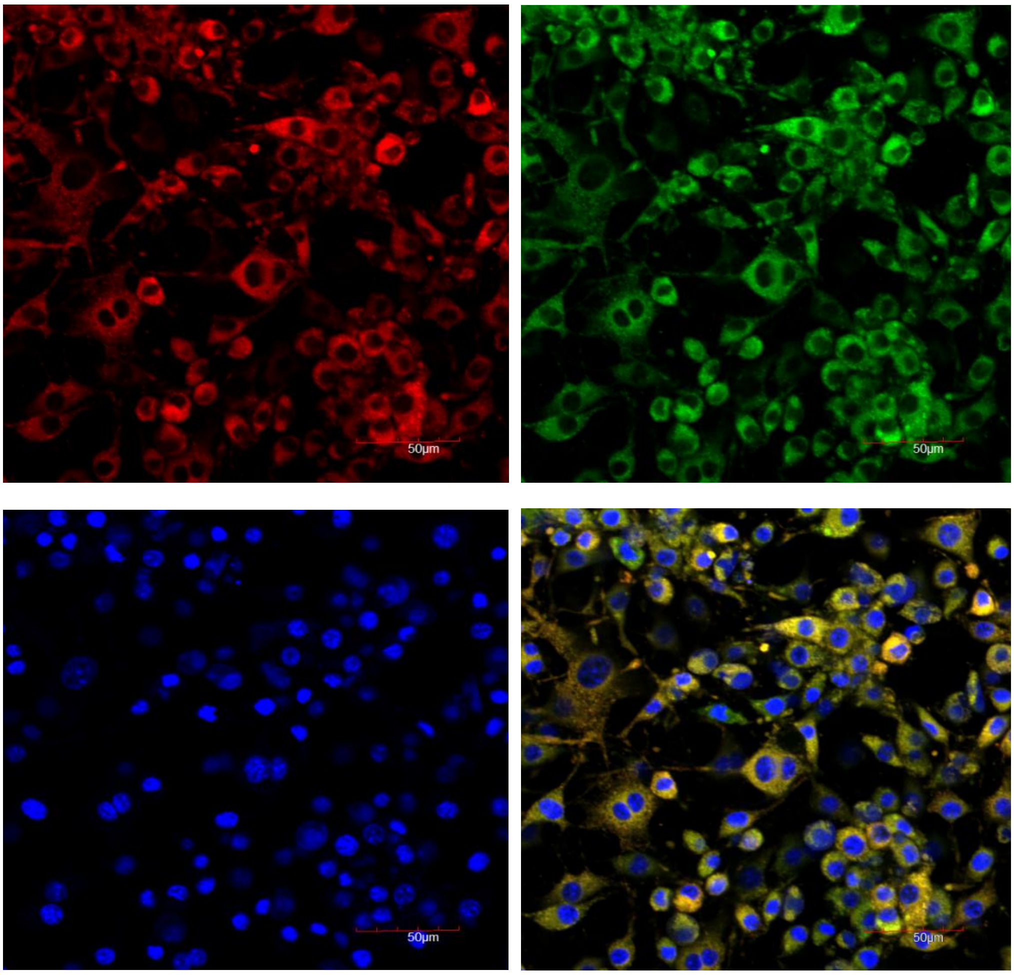

FAC 200

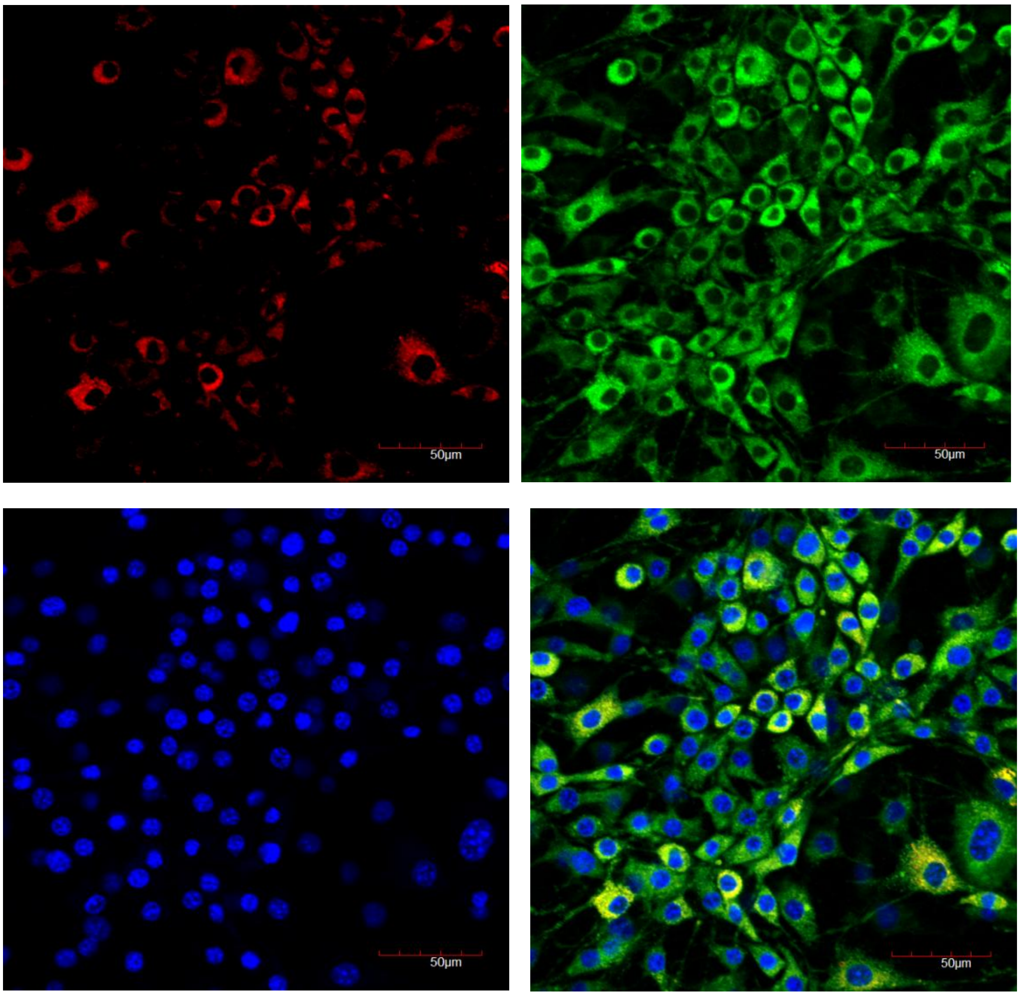

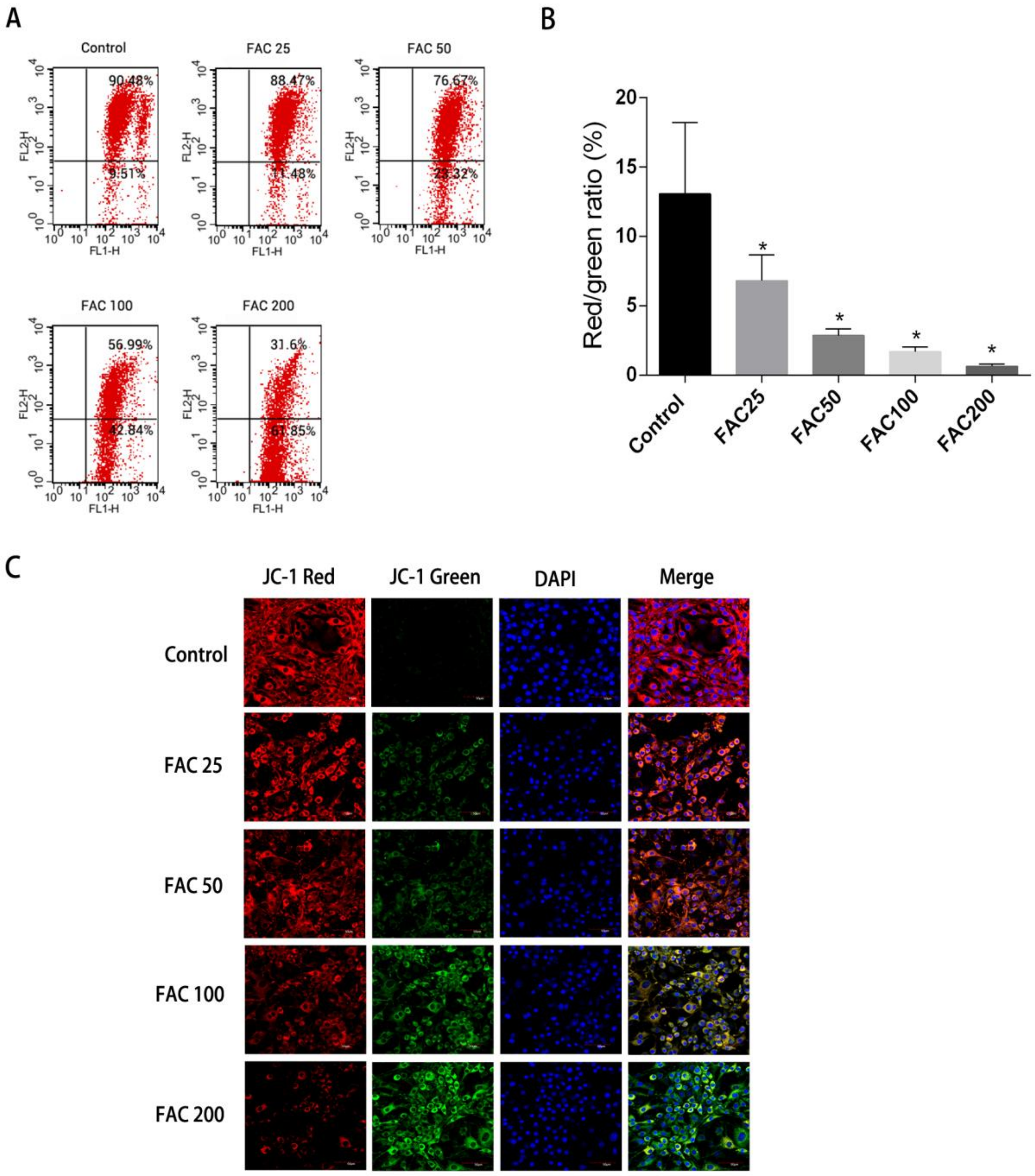
A
Control
90.48%
9.51%
FAC 25
88.47%
11.48%
FAC 50
76.67%
23.32%
FAC 100
56.99%
42.84%
FAC 200
31.6%
67.85%
FL2-H
FL1-H
B
Red/green ratio (%)
Control
FAC25
FAC50
FAC100
FAC200
C
JC-1 Red
JC-1 Green
DAPI
Merge
Control
FAC 25
FAC 50
FAC 100
FAC 200

Figure 8 Protective effects of NAC against iron-induced apoptosis.

ROS(DCFH-DA)	Control	NAC	FAC 200	FAC 200 + NAC
Group1				
No:	131106.001	131106.002	131106.004	131106.003
MFI(mean fluorescent intensity)	9.54	9.04	68.59	14.28
Group2				
No:	131016.002	131016.003	131016.005	131016.004
MFI(mean fluorescent intensity)	6.54	7.35	61.39	18.23
Group3				
No:	131217.002	131217.003	131217.005	131217.004
MFI(mean fluorescent intensity)	8.87	8.58	77.54	19.71

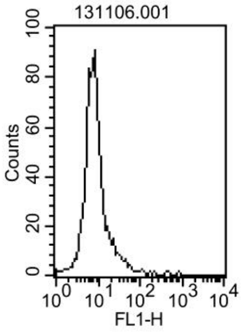

Marker	% Gated	Mean	Geo Mean
All	100.00	9.54	7.99

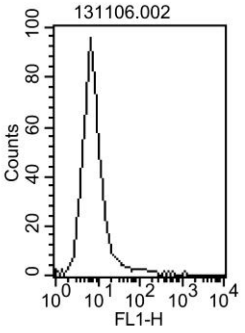

Marker	% Gated	Mean	Geo Mean
All	100.00	9.04	7.24

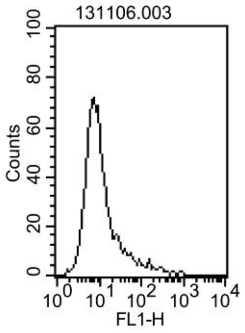

Marker	% Gated	Mean	Geo Mean
All	100.00	14.28	9.46

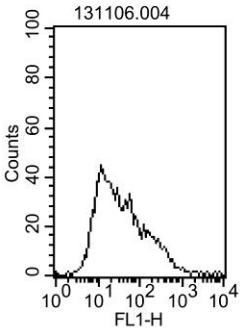

Marker	% Gated	Mean	Geo Mean
All	100.00	68.59	31.51

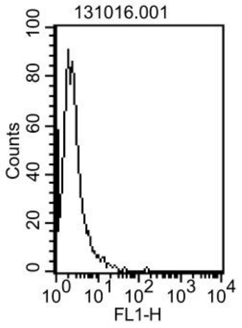

Marker	% Gated	Mean	Geo Mean
All	100.00	2.81	2.45

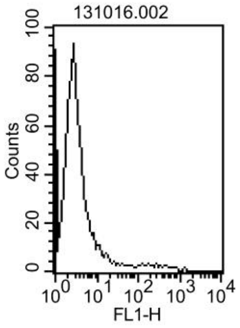

Marker	% Gated	Mean	Geo Mean
All	100.00	6.54	3.24

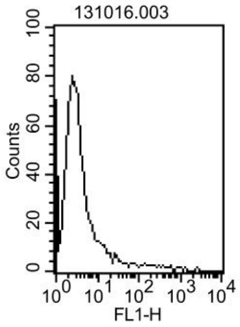

Marker	% Gated	Mean	Geo Mean
All	100.00	7.35	3.44

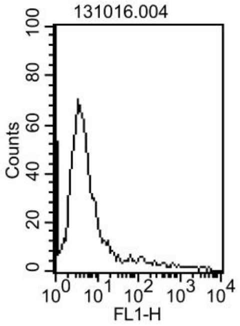

Marker	% Gated	Mean	Geo Mean
All	100.00	18.23	5.11

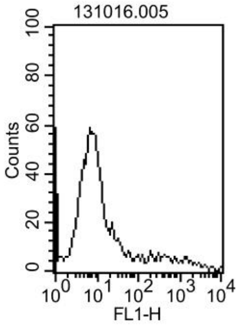

Marker	% Gated	Mean	Geo Mean
All	100.00	61.39	10.51

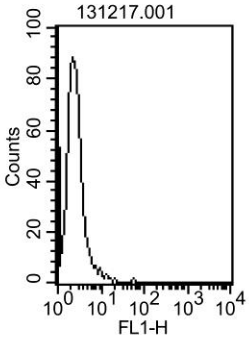

Marker	% Gated	Mean	Geo Mean
All	100.00	2.65	2.43

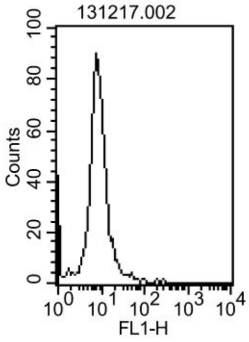

Marker	% Gated	Mean	Geo Mean
All	100.00	8.87	7.93

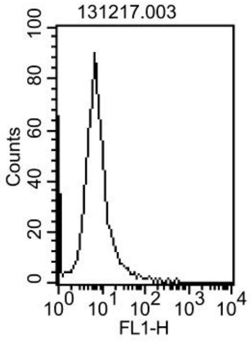

Marker	% Gated	Mean	Geo Mean
All	100.00	8.58	6.96

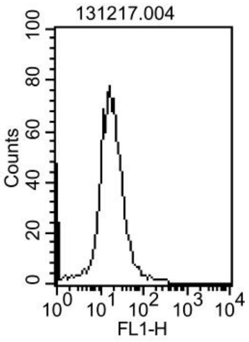

Marker	% Gated	Mean	Geo Mean
All	100.00	19.71	16.60

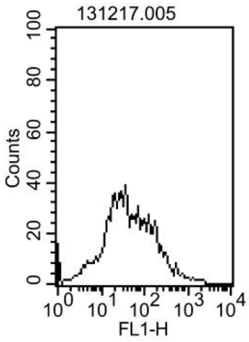

Marker	% Gated	Mean	Geo Mean
All	100.00	77.54	40.45

Mitochondrial membrane potential (MMP)	Control	NAC	FAC 200	FAC 200 + NAC
Group1				
No:	131217.01	131217.011	131217.012	131217.013
Red fluorescent intensity	93	95.83	38.91	76.14
Green fluorescent intensity	6.98	4.14	61.01	23.85
Group2				
No:	151023.001	151023.002	151023.003	151023.004
Red fluorescent intensity	92.02	91.53	36.18	77.72
Green fluorescent intensity	7.96	8.47	63.81	22.28
Group3				
No:	151027.001	151027.002	151027.003	151027.004
Red fluorescent intensity	93.57	92.28	30.73	78.33
Green fluorescent intensity	6.43	7.71	69.23	21.67

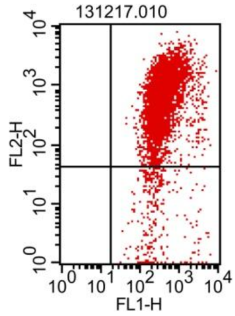

Quad	% Gated
UL	0.00
UR	93.00
LL	0.02
LR	6.98

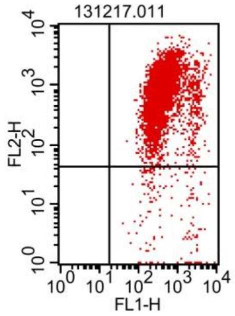

Quad	% Gated
UL	0.00
UR	95.83
LL	0.02
LR	4.14

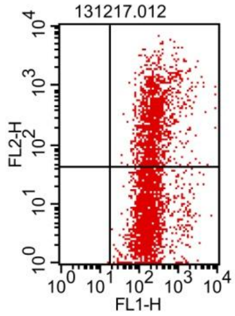

Quad	% Gated
UL	0.00
UR	38.91
LL	0.08
LR	61.01

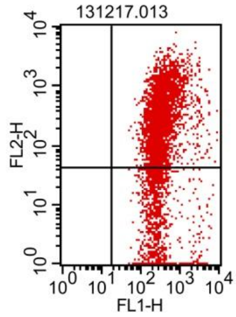

Quad	% Gated
UL	0.00
UR	76.14
LL	0.01
LR	23.85

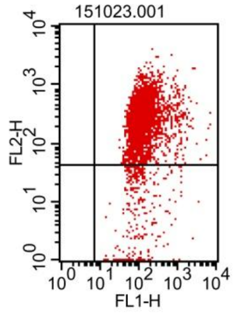

Quad	% Gated
UL	0.00
UR	92.02
LL	0.01
LR	7.96

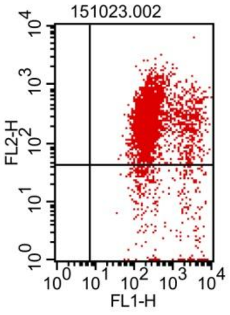

Quad	% Gated
UL	0.00
UR	91.53
LL	0.00
LR	8.47

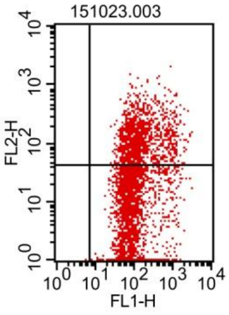

Quad	% Gated
UL	0.00
UR	36.18
LL	0.01
LR	63.81

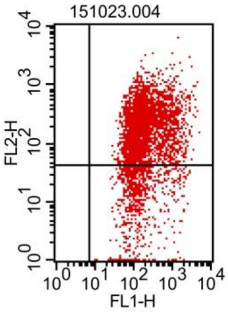

Quad	% Gated
UL	0.00
UR	77.72
LL	0.00
LR	22.28

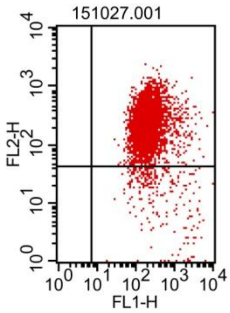

Quad	% Gated
UL	0.00
UR	93.57
LL	0.00
LR	6.43

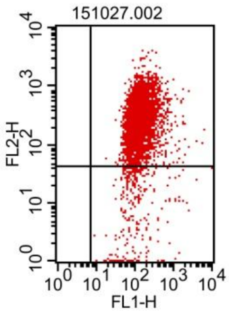

Quad	% Gated
UL	0.00
UR	92.28
LL	0.01
LR	7.71

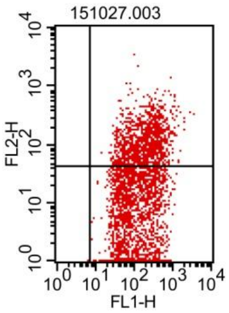

Quad	% Gated
UL	0.00
UR	30.73
LL	0.04
LR	69.23

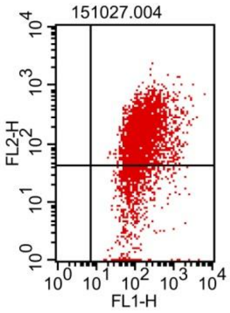

Quad	% Gated
UL	0.00
UR	78.33
LL	0.00
LR	21.67

Apoptosis rate(%)	Control	NAC	FAC 200	FAC 200 + NAC
Group1				
No:	131121.001	131121.006	131121.005	131121.007
Early apoptosis	1.04	0.73	17.9	6.49
Late apoptosis	3.31	2.56	36.02	6.63
Apoptosis rate(%)	4.35	3.29	53.92	13.12
Group2				
No:	131202.001	131202.006	131202.005	131202.007
Early apoptosis	0.91	0.95	27.69	4.77
Late apoptosis	3.06	2.54	35.89	6.37
Apoptosis rate(%)	3.97	3.49	63.58	11.14
Group3				
No:	131104.001	131104.006	131104.005	131104.007
Early apoptosis	0.65	0.55	15.18	2.61
Late apoptosis	3.46	1.08	37.48	13.93
Apoptosis rate(%)	4.11	1.63	52.66	16.54

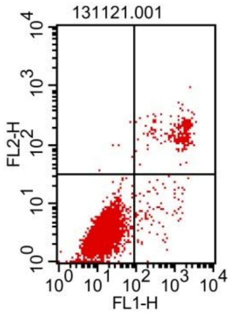

Quad	% Gated
UL	0.07
UR	3.31
LL	95.59
LR	1.04

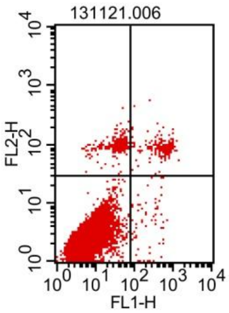

Quad	% Gated
UL	4.41
UR	2.56
LL	92.30
LR	0.73

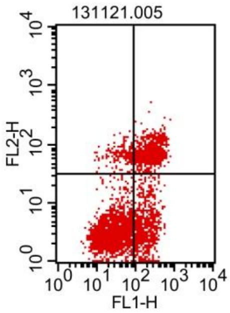

Quad	% Gated
UL	4.88
UR	36.02
LL	41.20
LR	17.90

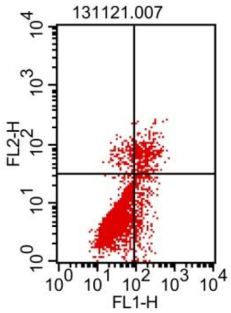

Quad	% Gated
UL	2.83
UR	6.63
LL	84.05
LR	6.49

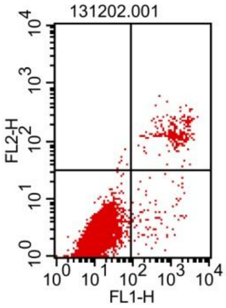

Quad	% Gated
UL	0.07
UR	3.06
LL	95.97
LR	0.91

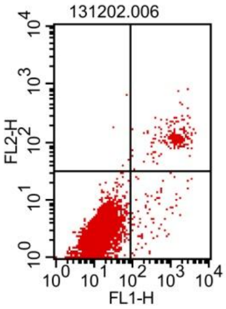

Quad	% Gated
UL	0.06
UR	2.54
LL	96.44
LR	0.95

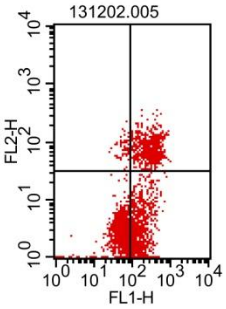

Quad	% Gated
UL	2.02
UR	35.89
LL	34.40
LR	27.69

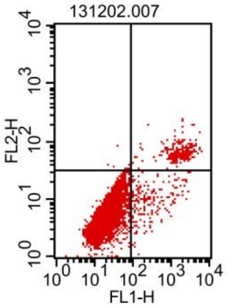

Quad	% Gated
UL	0.25
UR	6.37
LL	88.61
LR	4.77

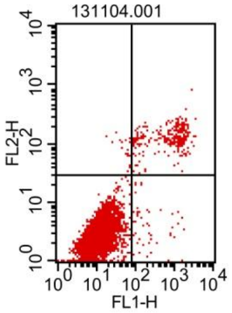

Quad	% Gated
UL	0.29
UR	3.46
LL	95.60
LR	0.65

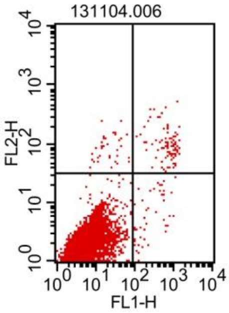

Quad	% Gated
UL	0.42
UR	1.08
LL	97.94
LR	0.55

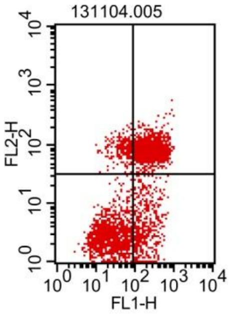

Quad	% Gated
UL	8.30
UR	37.48
LL	39.03
LR	15.18

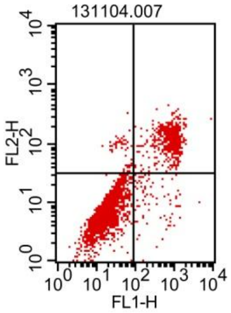

Quad	% Gated
UL	1.70
UR	13.93
LL	81.76
LR	2.61

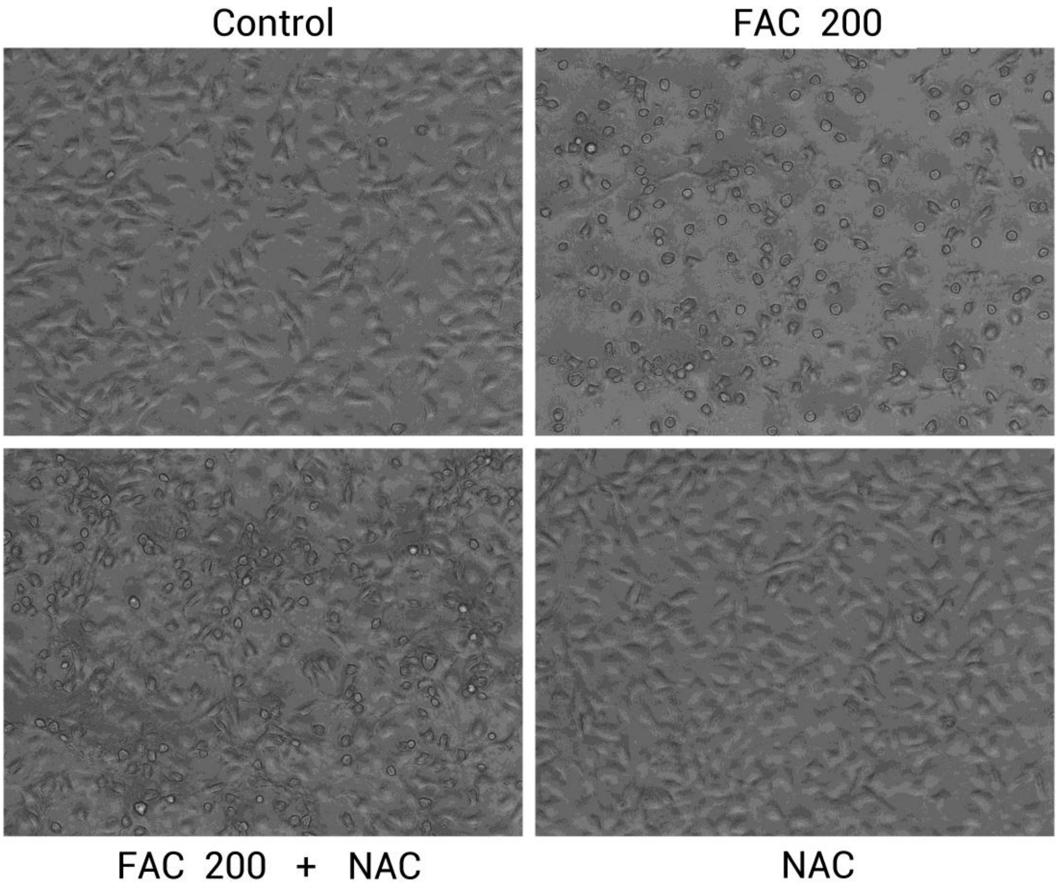
Control
FAC 200
FAC 200 + NAC
NAC

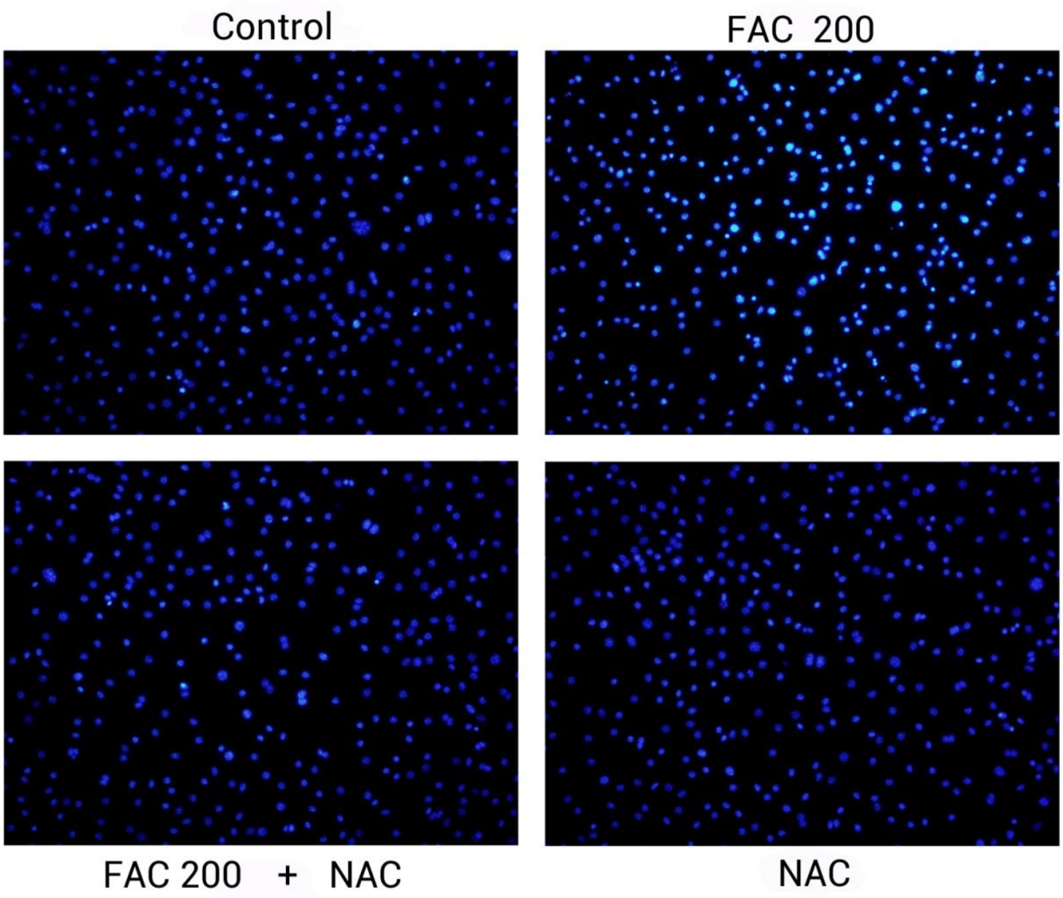

Control
FAC 200
FAC 200 + NAC
NAC

WB

Beta-actin Control NAC FAC 200 FAC 200 + NAC

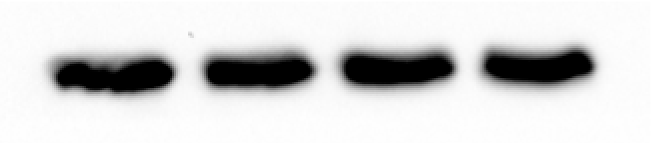

Bcl-2

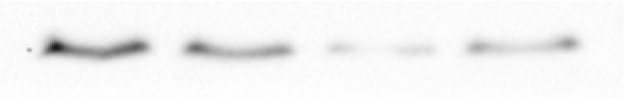

Bax

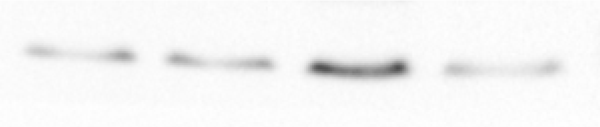

Cleaved-caspase3

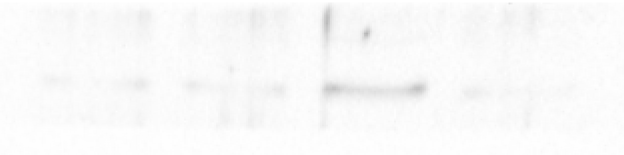

Cyto c

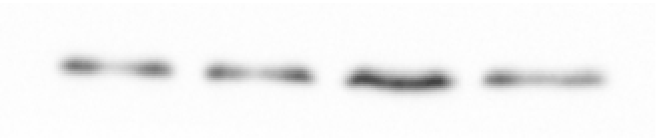

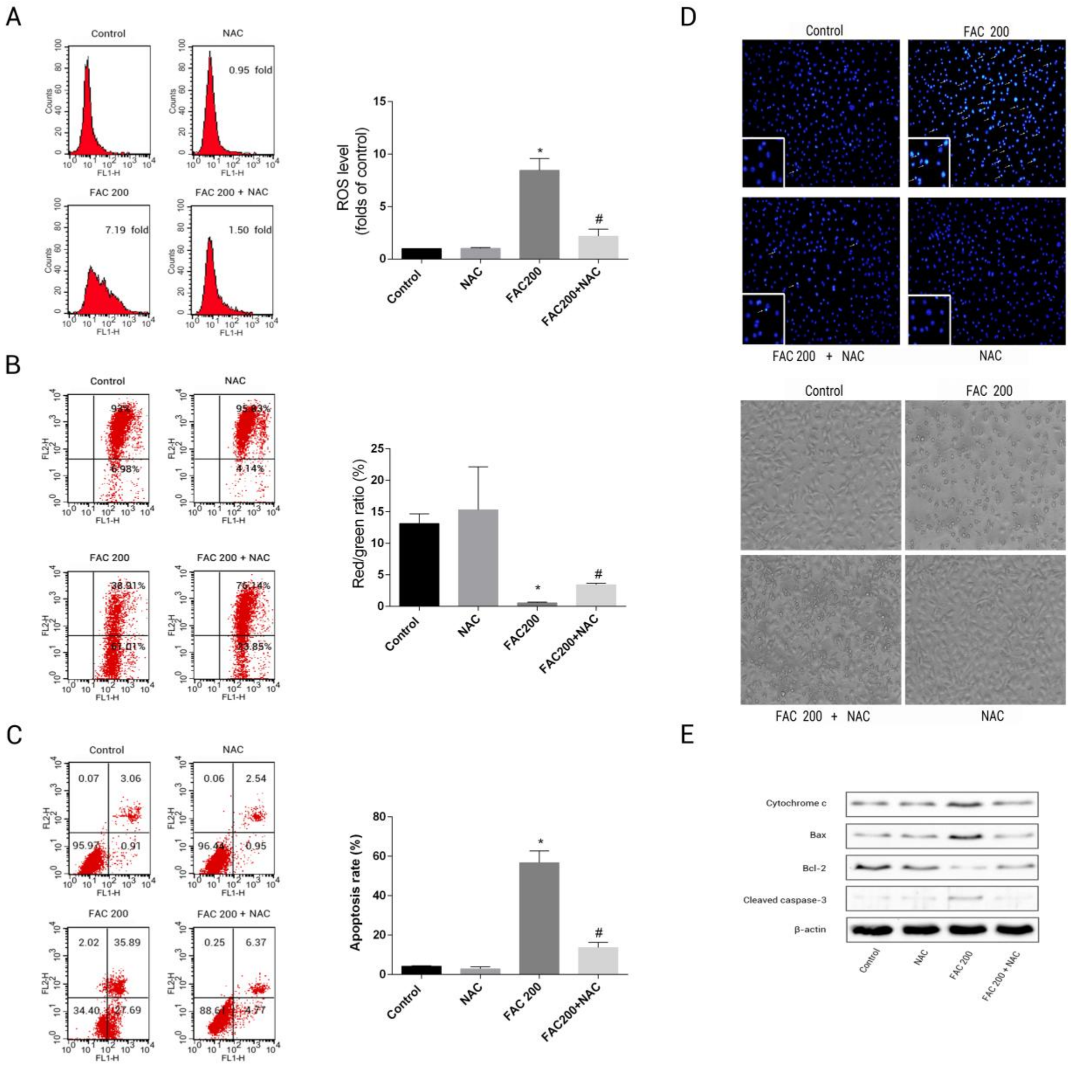
A
Control
NAC
0.95 fold
FAC 200
7.19 fold
FAC 200 + NAC
1.50 fold
Counts
FL1-H
ROS level
(folds of control)
Control
NAC
FAC200
FAC200+NAC
B
Control
NAC
FAC 200
FAC 200 + NAC
FL2-H
FL1-H
Red/green ratio (%)
C
Control
0.07
3.06
95.97
0.91
NAC
0.06
2.54
0.95
FAC 200
2.02
35.89
34.40
FAC 200 + NAC
0.25
6.37
Apoptosis rate (%)
FAC 200
D
Control
FAC 200
FAC 200 + NAC
NAC
E
Cytochrome c
Bax
Bcl-2
Cleaved caspase-3
β-actin
Control
NAC
FAC 200
FAC 200 + NAC

Figure 9 Cytotoxic effects of iron on the viability of bone marrow-derived MSCs.

24h					
	Control	FAC 25	FAC 50	FAC 100	FAC 200
Group1	0.509	0.513	0.519	0.535	0.528
	0.517	0.568	0.536	0.516	0.512
	0.518	0.576	0.532	0.53	0.544
Group2	0.499	0.502	0.531	0.561	0.553
	0.522	0.516	0.529	0.522	0.512
	0.534	0.544	0.533	0.508	0.523
Group3	0.601	0.599	0.596	0.593	0.588
	0.589	0.59	0.594	0.586	0.595
	0.576	0.591	0.6	0.574	0.579

72h					
	Control	FAC 25	FAC 50	FAC 100	FAC 200
Group1	1.015	0.943	0.813	0.656	0.671
	1.029	0.996	0.795	0.632	0.615
	1.124	0.987	0.762	0.613	0.631
Group2	0.96	0.902	0.782	0.603	0.57
	0.988	0.897	0.766	0.59	0.576
	0.979	0.897	0.755	0.583	0.599
Group3	0.997	0.883	0.832	0.682	0.483
	1.011	0.916	0.832	0.642	0.516
	1.054	0.932	0.816	0.65	0.513

144h					
	Control	FAC 25	FAC 50	FAC 100	FAC 200
Group1	1.53	1.473	1.363	1.09	0.727
	1.478	1.43	1.338	1.032	0.788
	1.464	1.43	1.332	1.036	0.687
Group2	1.558	1.564	1.452	1.178	0.953
	1.53	1.527	1.413	1.189	0.897
	1.535	1.48	1.331	1.208	0.839
Group3	1.498	1.428	1.321	1.007	0.776
	1.483	1.449	1.369	1.062	0.677
	1.518	1.389	1.301	1.062	0.791

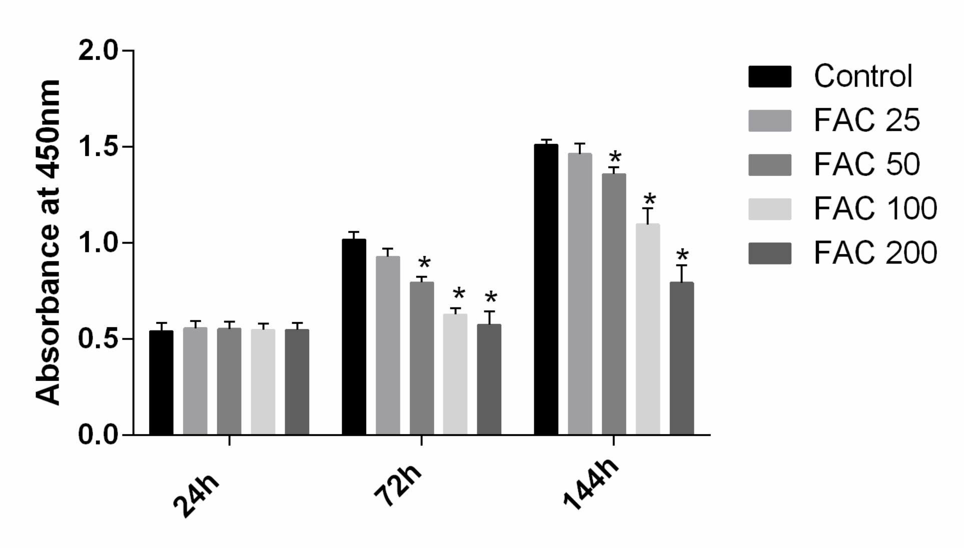

Figure 10 Iron induced apoptosis in bone marrow-derived MSCs.

Apoptosis rate(%)	Control	FAC 200
Group1		
No:	160724.001	160724.002
Early apoptosis	2.42	4.46
Late apoptosis	2.15	24.97
Group2		
No:	160724.003	160724.004
Early apoptosis	2.4	5.88
Late apoptosis	5.85	18.65
Group3		
No:	160724.005	160724.006
Early apoptosis	5.58	7.53
Late apoptosis	2.12	24.66

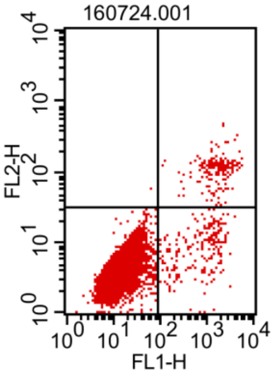

Quad	% Gated
UL	0.01
UR	2.15
LL	95.41
LR	2.42

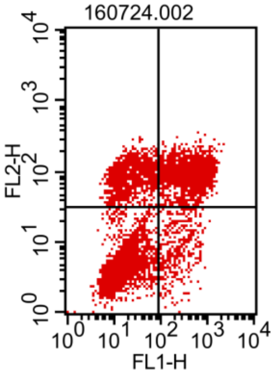

Quad	% Gated
UL	15.00
UR	24.97
LL	55.57
LR	4.46

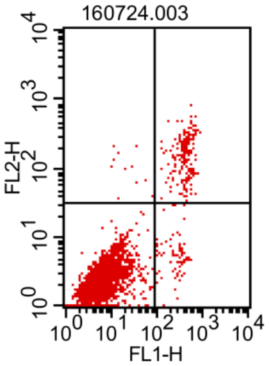

Quad	% Gated
UL	0.36
UR	5.85
LL	91.39
LR	2.40

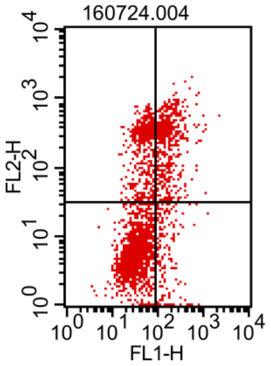

Quad	% Gated
UL	16.07
UR	18.65
LL	59.41
LR	5.88

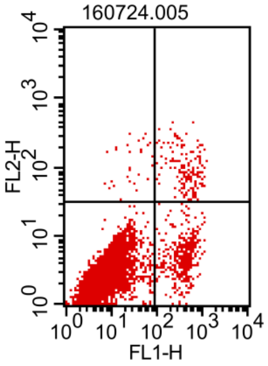

Quad	% Gated
UL	0.39
UR	2.12
LL	91.91
LR	5.58

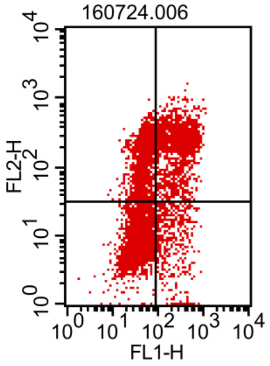

Quad	% Gated
UL	26.85
UR	24.66
LL	40.96
LR	7.53

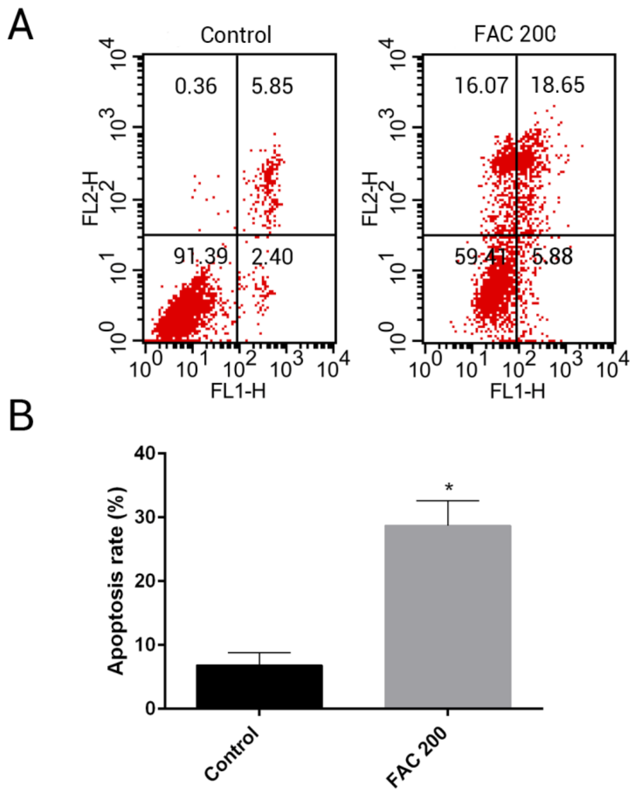
A
Control
FAC 200
0.36
5.85
91.39
2.40
16.07
18.65
59.41
5.88
FL2-H
FL1-H
B
Apoptosis rate (%)
40
30
20
10
0
*
Control
FAC 200

Figure 11 Effect of iron on ALP activity and matrix calcification

ALP activity (ng/mg protein)					
	Control	FAC 25	FAC 50	FAC 100	FAC 200
Group1	99.8	72.3	56.5	30.2	22.1
Group2	89.6	60.1	46.4	25.8	26.3
Group3	101.2	61.3	59.2	24.6	25.2

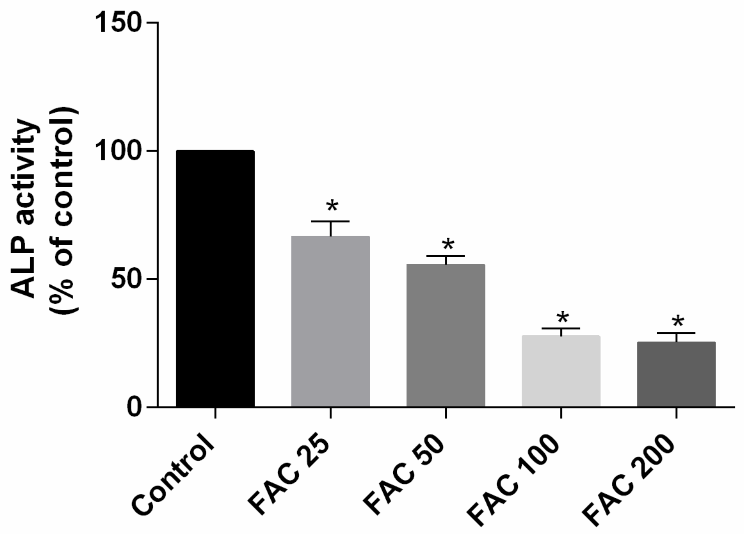

Control

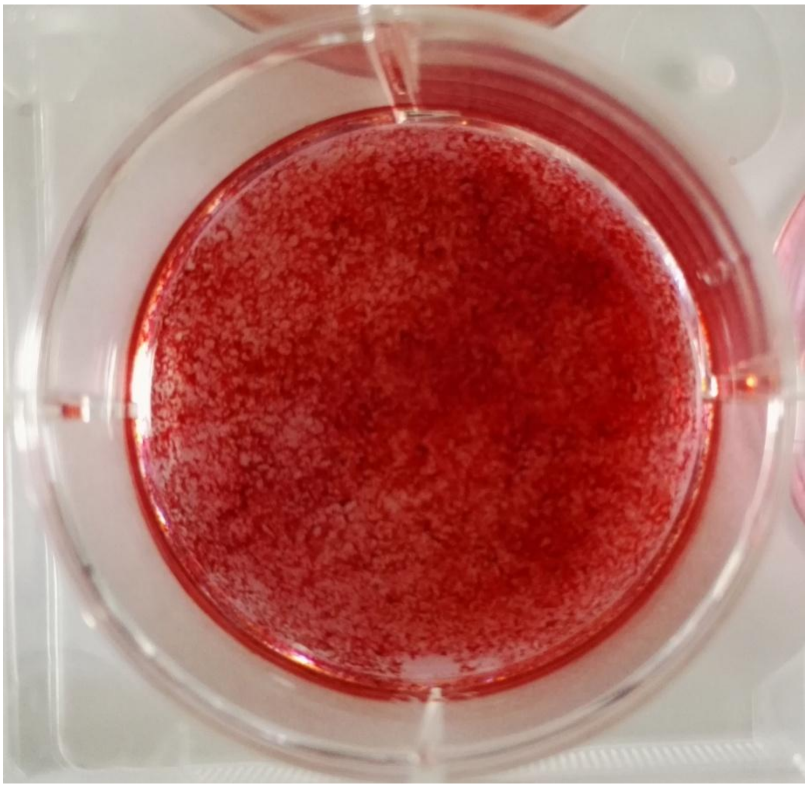

FAC 25

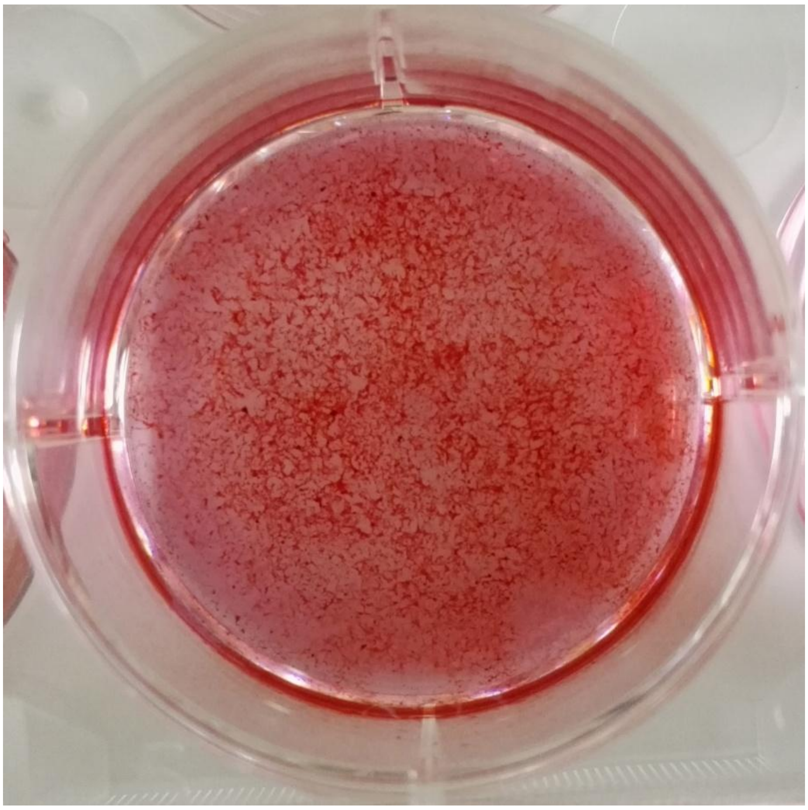

FAC 50

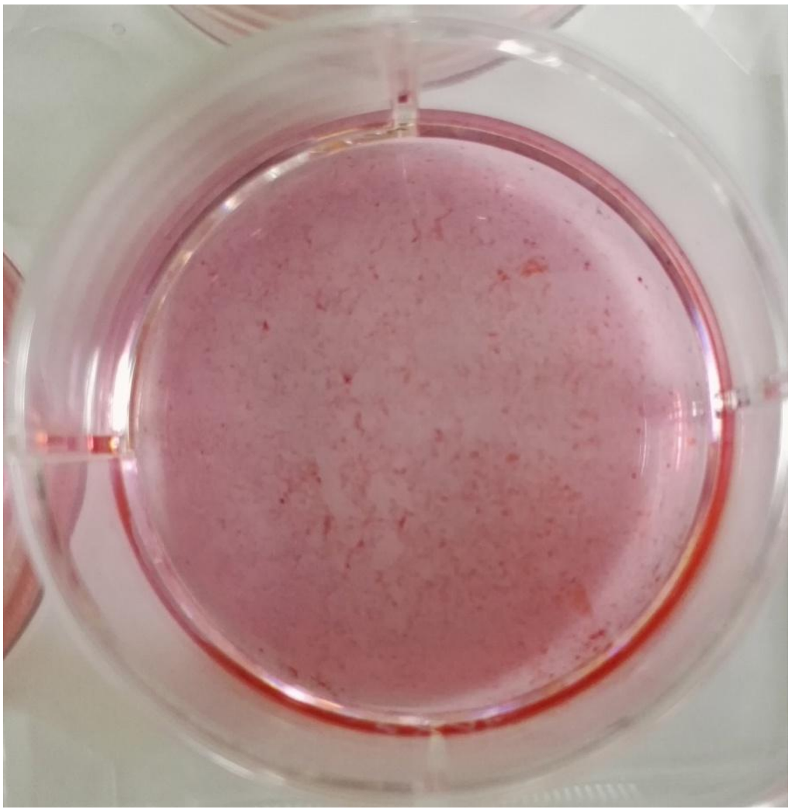

FAC 100

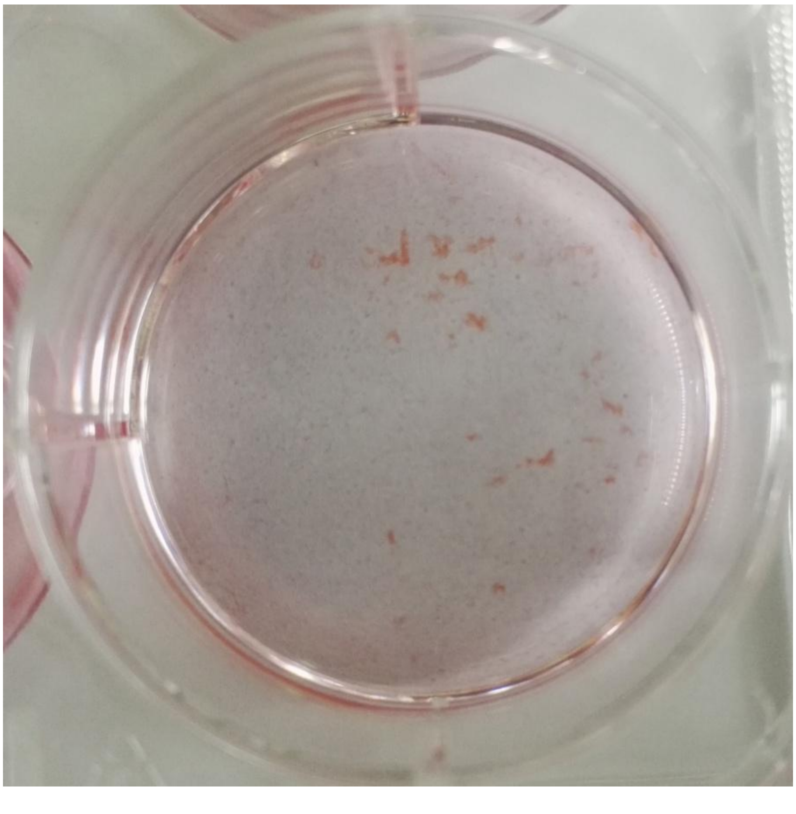

FAC 200

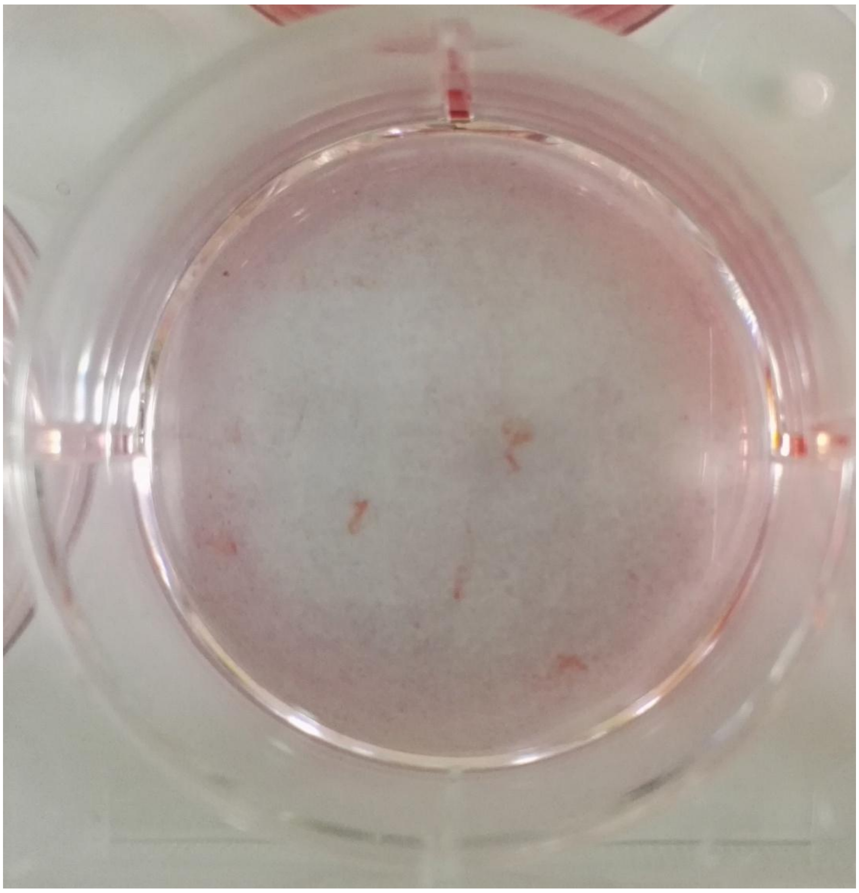

Control

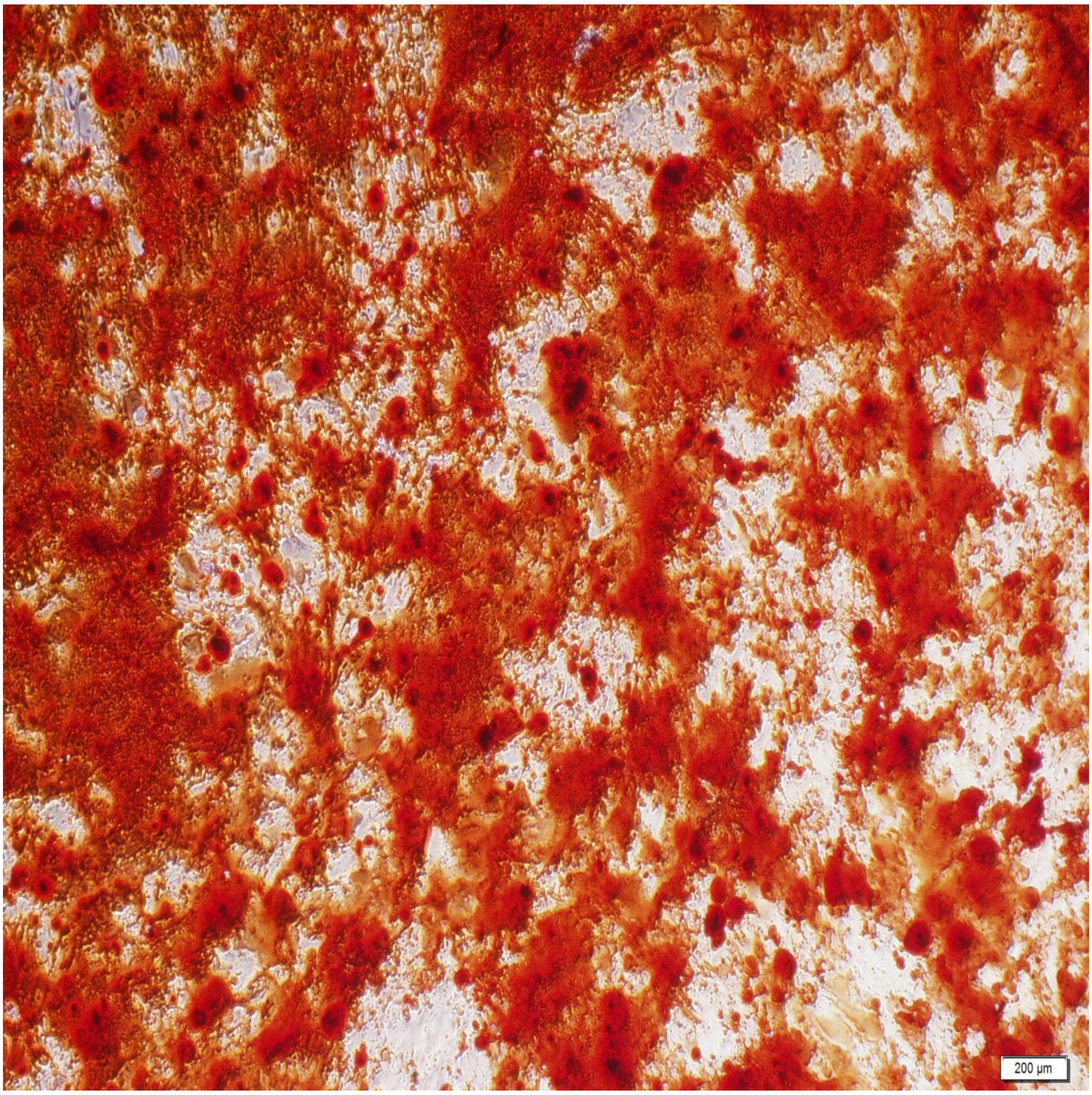

FAC 25

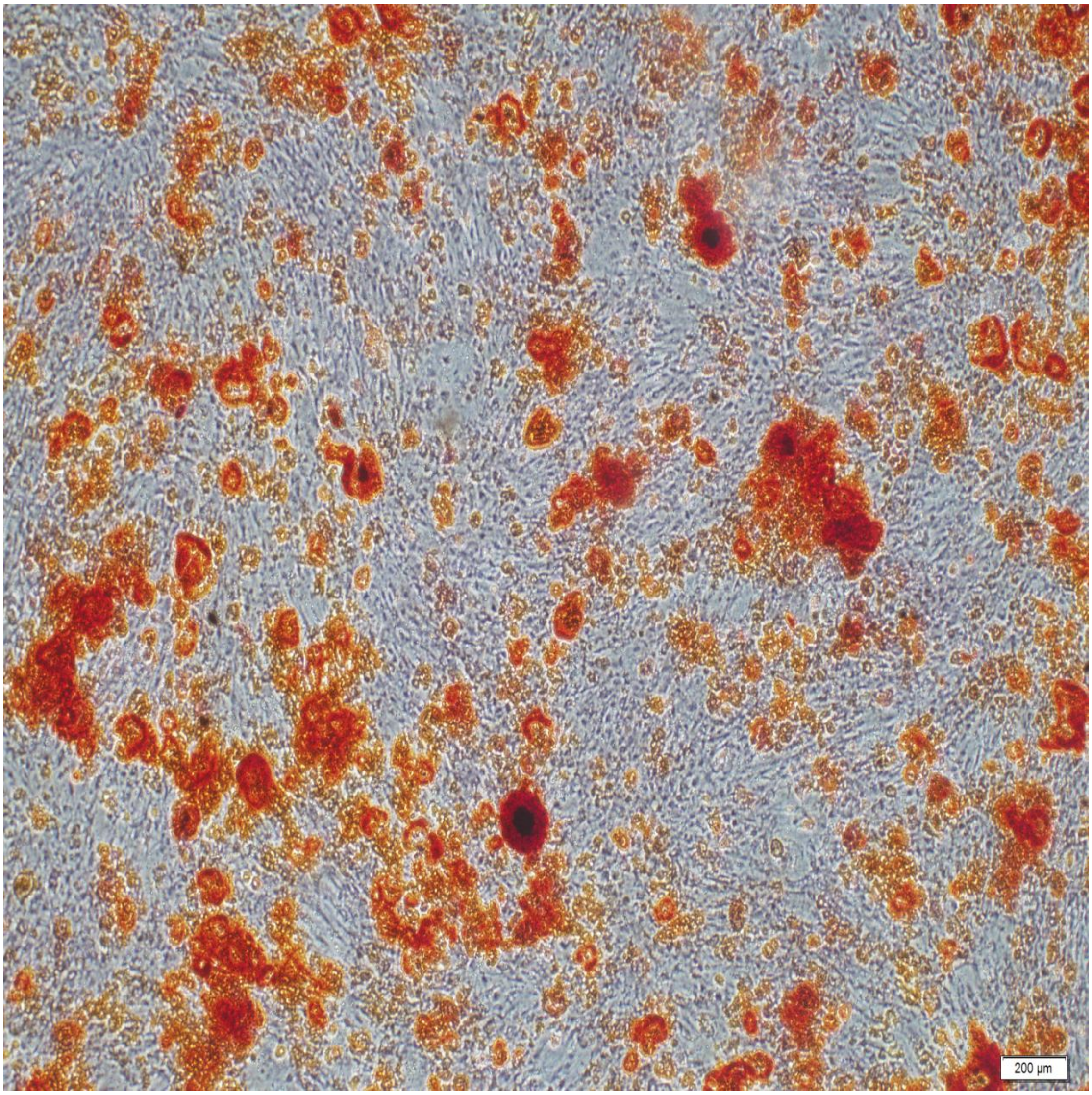

FAC 50

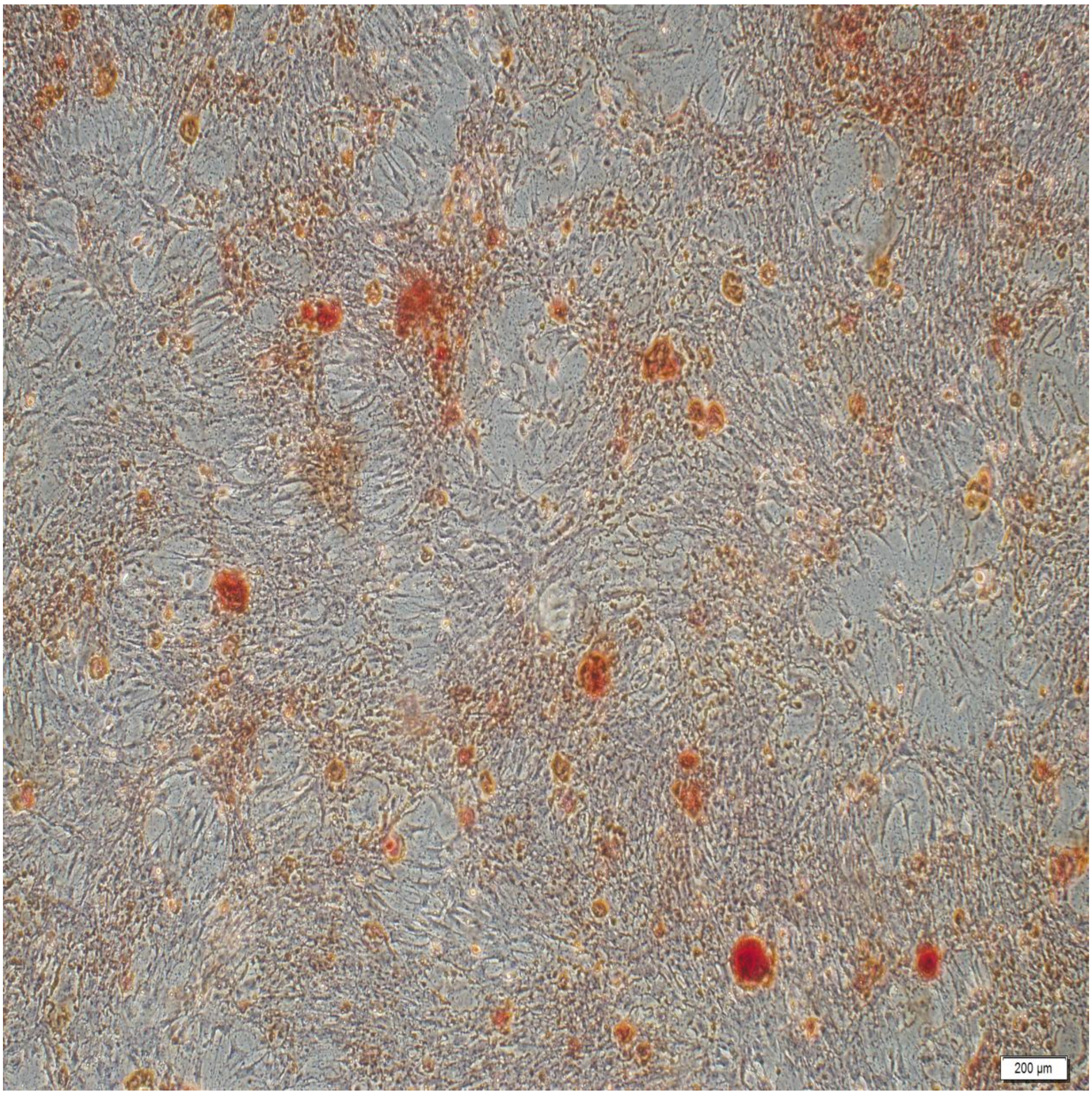

FAC 100

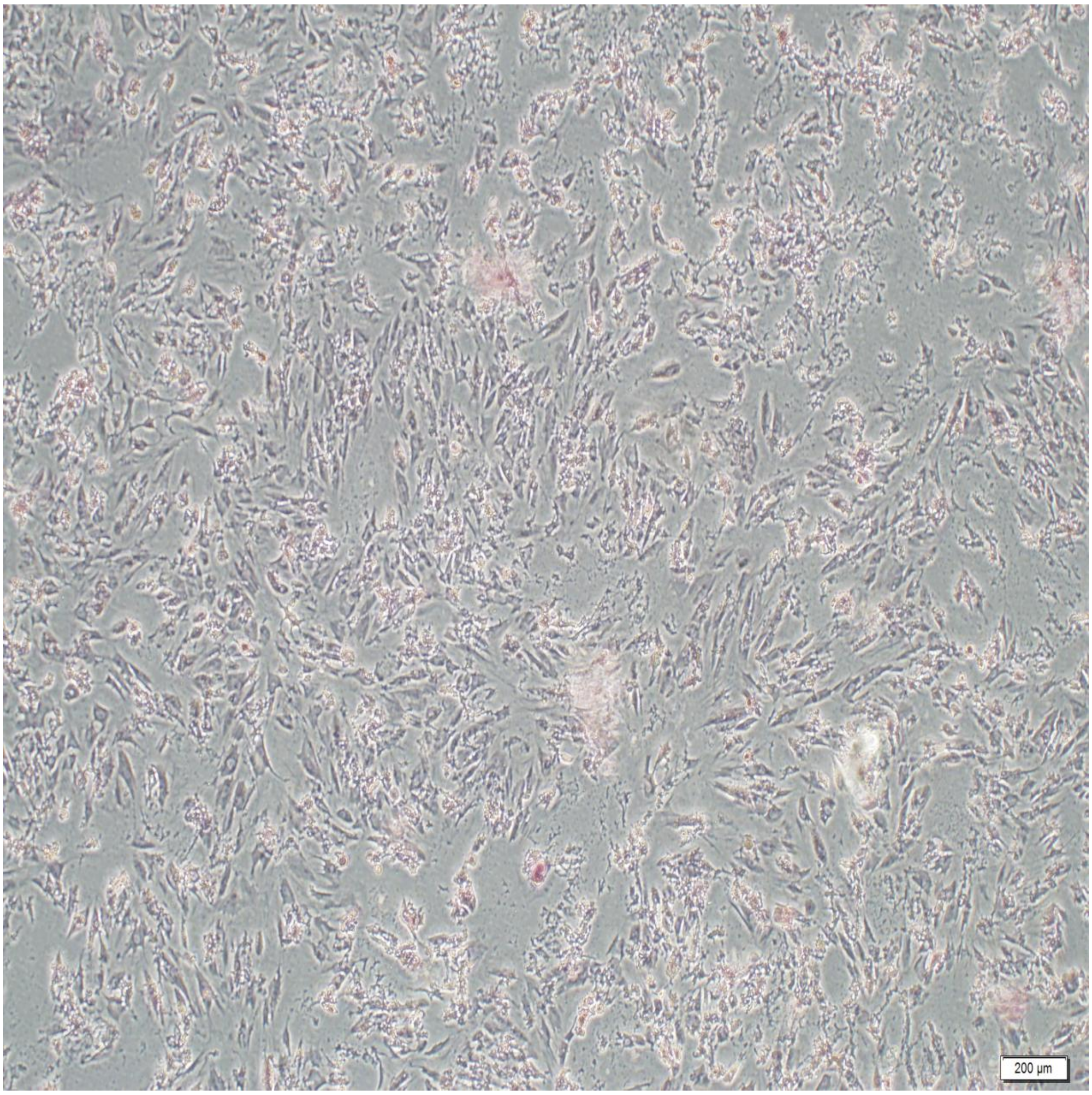

FAC 200

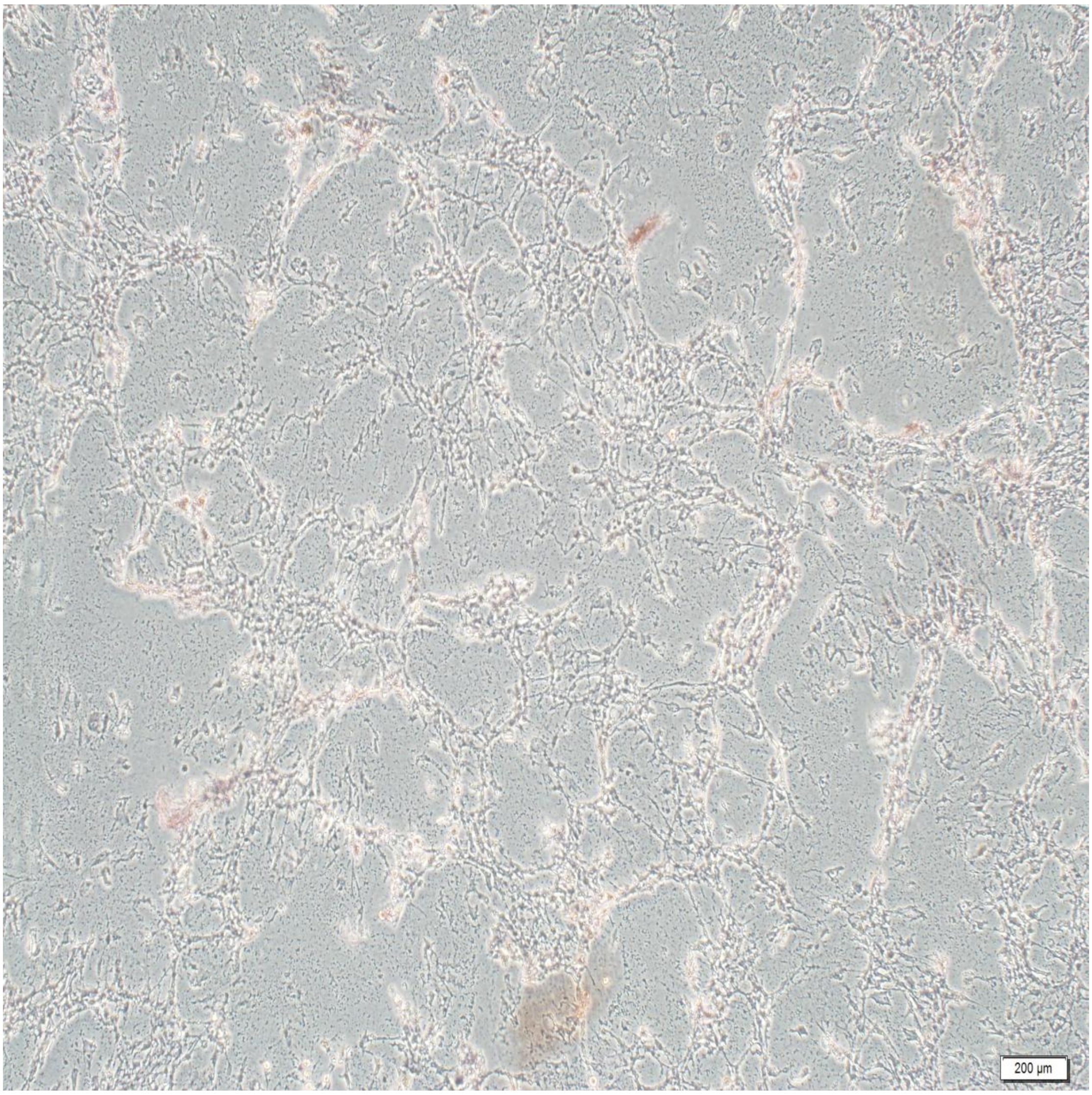

ARS(OD)					
	Control	FAC 25	FAC 50	FAC 100	FAC 200
Group1	1.868	0.914	0.418	0.213	0.226
Group2	1.984	0.869	0.506	0.234	0.206
Group3	1.894	0.938	0.494	0.268	0.247

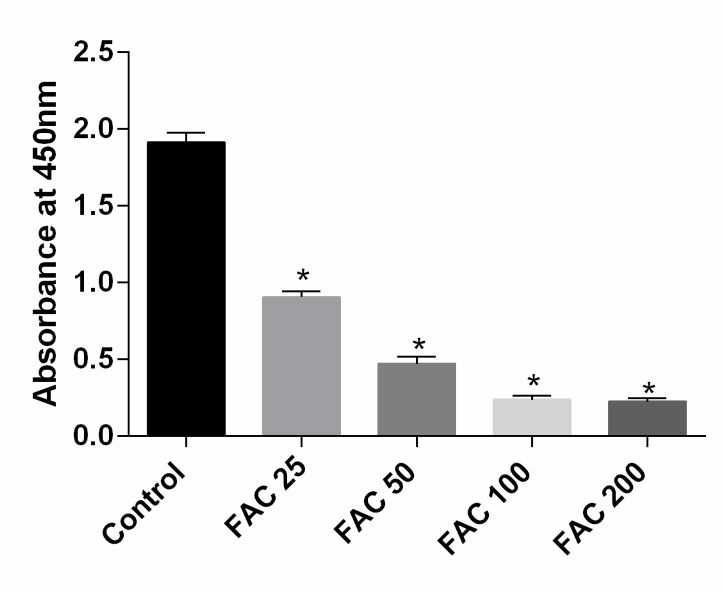

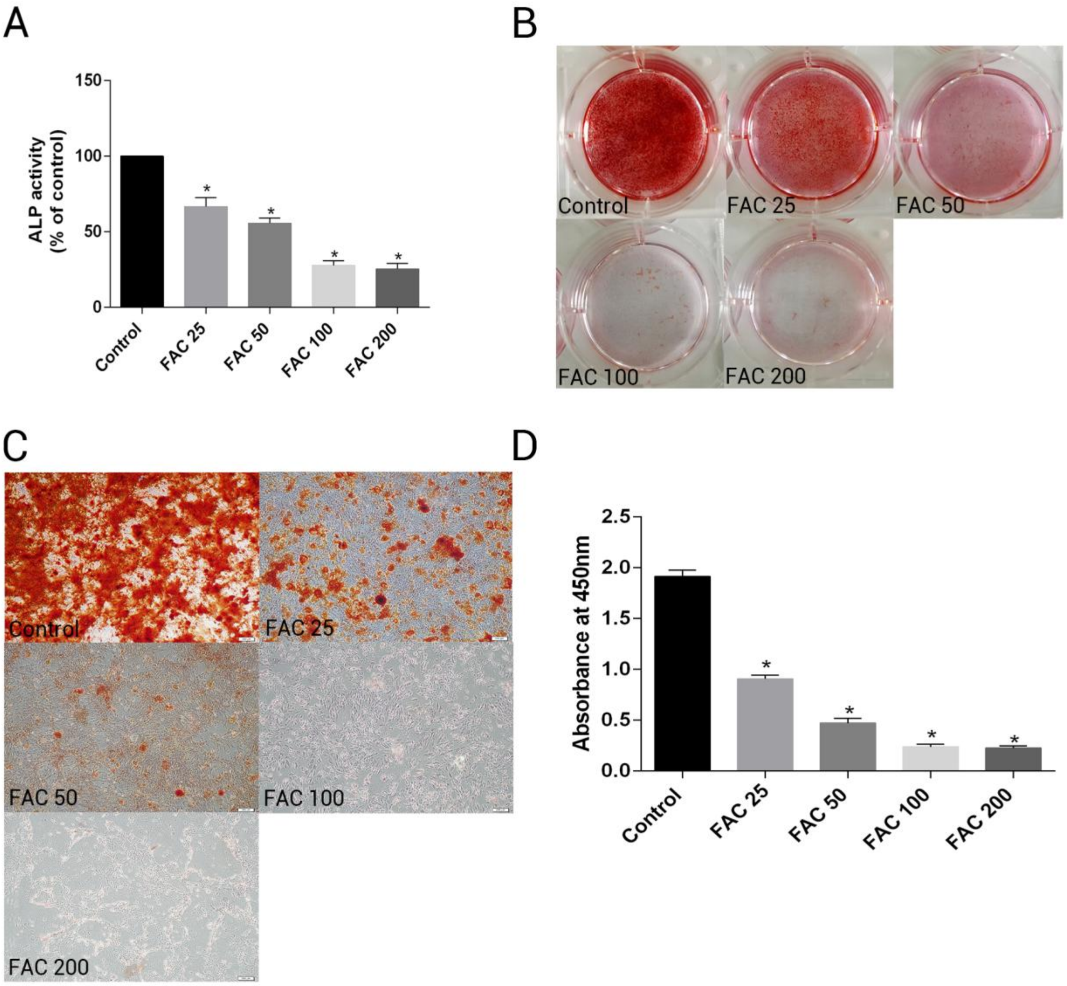
A
ALP activity
(% of control)
150
100
50
0
*
Control
FAC 25
FAC 50
FAC 100
FAC 200
B
Control
FAC 25
FAC 50
FAC 100
FAC 200
C
Control
FAC 25
FAC 50
FAC 100
FAC 200
D
Absorbance at 450nm
2.5
2.0
1.5
1.0
0.5
0.0
Control
FAC 25
FAC 50
FAC 100
FAC 200
