# Supplementary figures and images for: Iron overload induced death of osteoblasts in vitro: involvement of the mitochondrial apoptotic pathway
Source: PeerJ. 2016 Nov 8;4:e2611. doi: 10.7717/peerj.2611 (PMC5103817; doi:10.7717/peerj.2611)

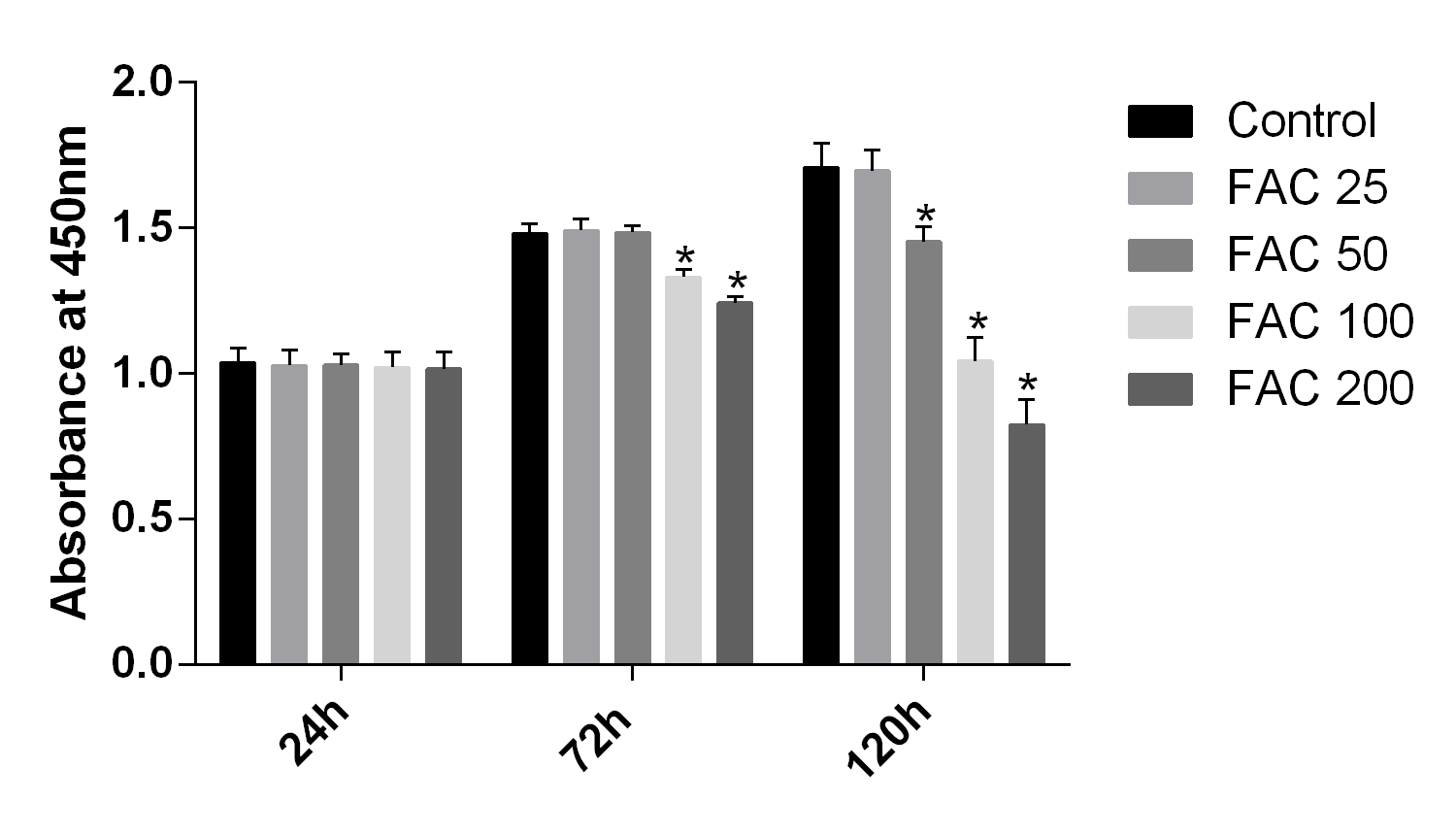

Supplement: Figure S1 — Viability of osteoblasts was evaluated by CCK-8 assay after treatment with FAC (25–200 µM) for 24 h, 72 h and 120 h. Compared to the control (FAC 0 µM), iron significantly reduced cell viability in a dose-dependent manner after 120-h FAC treatment. The values are presented as means ± SD, n = 3; 793 ∗P < 0.05 vs. the control. [file peerj-04-2611-s002.png]

**A**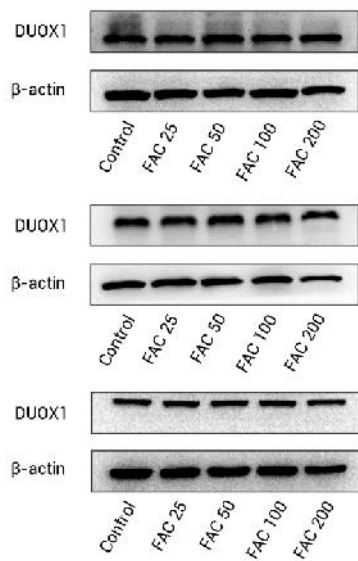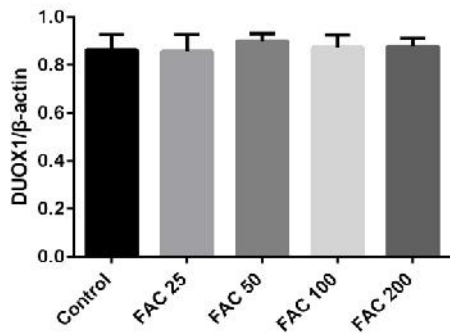**B**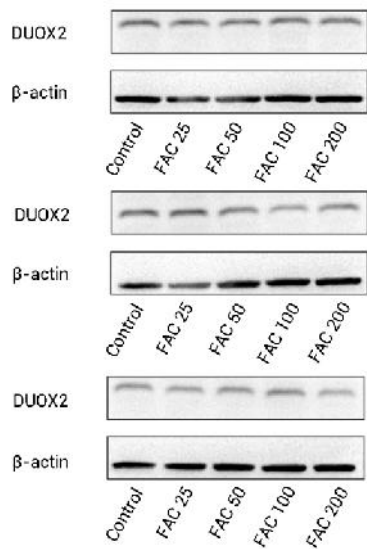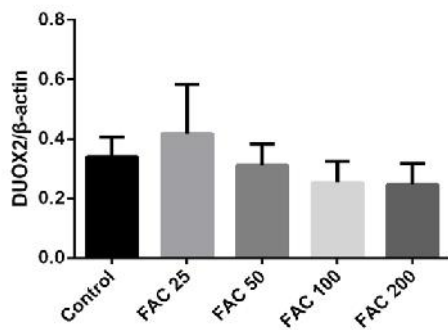

Supplement: Figure S2 — (A-B) Representative western blot data for DUOX1 and DUOX2 in osteoblasts following exposure to FAC (0–200 µM) for 120 h. β-actin was used as an internal control. Date are presented as means ± SD, n = 3; ∗P < 0.05 vs. the control. [file peerj-04-2611-s003.pdf]
